# Metabolic pathways engineering for drought or/and heat tolerance in cereals

**DOI:** 10.3389/fpls.2023.1111875

**Published:** 2023-09-22

**Authors:** Songtao Liu, Tinashe Zenda, Zaimin Tian, Zhihong Huang

**Affiliations:** ^1^Hebei Key Laboratory of Quality & Safety Analysis-Testing for Agro-Products and Food, Hebei North University, Zhangjiakou, China; ^2^State Key Laboratory of North China Crop Improvement and Regulation, Hebei Agricultural University, Baoding, China

**Keywords:** D/+H stress, metabolic pathway, synthetic biology, pathways crosstalk, cereal crops, multiple-trait modification

## Abstract

Drought (D) and heat (H) are the two major abiotic stresses hindering cereal crop growth and productivity, either singly or in combination (D/+H), by imposing various negative impacts on plant physiological and biochemical processes. Consequently, this decreases overall cereal crop production and impacts global food availability and human nutrition. To achieve global food and nutrition security *vis-a-vis* global climate change, deployment of new strategies for enhancing crop D/+H stress tolerance and higher nutritive value in cereals is imperative. This depends on first gaining a mechanistic understanding of the mechanisms underlying D/+H stress response. Meanwhile, functional genomics has revealed several stress-related genes that have been successfully used in target-gene approach to generate stress-tolerant cultivars and sustain crop productivity over the past decades. However, the fast-changing climate, coupled with the complexity and multigenic nature of D/+H tolerance suggest that single-gene/trait targeting may not suffice in improving such traits. Hence, in this review-cum-perspective, we advance that targeted multiple-gene or metabolic pathway manipulation could represent the most effective approach for improving D/+H stress tolerance. First, we highlight the impact of D/+H stress on cereal crops, and the elaborate plant physiological and molecular responses. We then discuss how key primary metabolism- and secondary metabolism-related metabolic pathways, including carbon metabolism, starch metabolism, phenylpropanoid biosynthesis, *γ-*aminobutyric acid (GABA) biosynthesis, and phytohormone biosynthesis and signaling can be modified using modern molecular biotechnology approaches such as CRISPR-Cas9 system and synthetic biology (Synbio) to enhance D/+H tolerance in cereal crops. Understandably, several bottlenecks hinder metabolic pathway modification, including those related to feedback regulation, gene functional annotation, complex crosstalk between pathways, and metabolomics data and spatiotemporal gene expressions analyses. Nonetheless, recent advances in molecular biotechnology, genome-editing, single-cell metabolomics, and data annotation and analysis approaches, when integrated, offer unprecedented opportunities for pathway engineering for enhancing crop D/+H stress tolerance and improved yield. Especially, Synbio-based strategies will accelerate the development of climate resilient and nutrient-dense cereals, critical for achieving global food security and combating malnutrition.

## 1 Introduction

Crop plants` sedentary nature exposes them to persistent environmental and pathogenic stresses, often causing harmful effects (Ahanger et al., 2017; Iqbal et al., 2021b; Zhang et al., 2022a). Among several abiotic stress factors, drought (DS) or/and heat stress (HS) hinder plant fitness, growth and productivity the most (Lamaoui et al., 2018; Tenorio Berrío et al., 2022). Conspicuously, the unequivocal climate change intensifies the intensities, durations and incidences of D+H stress across spatiotemporal scales (Dai, 2013; Environment, U. N 2021; Zandalinas et al., 2021a). This aggravates the adverse impacts on cereal crops such as wheat (*Triticum aestivum*), rice (*Oryza sativa*), maize (*Zea mays*), barley (*Hordeum vulgare*) and sorghum (*Sorghum bicolor*) across most terrestrial regions (Dhankher and Foyer, 2018; Santini et al., 2022), consequently fuelling global food and nutrition insecurities (Raza et al., 2019).

Particularly, the occurrence of D/+H stress at the reproductive stage has more devastating effects than at any other phenological stage in cereal crops (Barnabás et al., 2008; Sehgal et al., 2018). Besides, the combinatorial effects of D+H are huge than each individual stress effects compared (Zandalinas et al., 2018; Sinha et al., 2021). Therefore, biotechnological approaches that emphasize the development of transgenic crops under conditions mimicking field situations and focusing on the plant reproductive stage will significantly increase the opportunity of producing stress tolerant crops. Especially, developing customized cereal crops harbouring D/+H tolerance is critical for climate change resilience and food security attainment (Zhang et al., 2018; Rivero et al., 2022).

Over the past decades, coupling conventional plant breeding to modern approaches such as genomics assisted breeding (GAB) (Kole et al., 2015; Raza et al., 2021; Varshney et al., 2021b; Yadav et al., 2021), omics (Scossa et al., 2021; Zenda et al., 2021b; Singh et al., 2022), genetic engineering (Krenek et al., 2015), biotechnology (Dwivedi et al., 2020; Munaweera et al., 2022), and genome editing (Chen et al., 2019; Gao, 2021) has helped us decode the multi-level nature of plant responses to abiotic stresses, identify key genetic factors modulating complex plant stress-regulatory networks, and introgress beneficial traits, leading to practical applications in stress tolerance and quality improvement in crops (Scheben et al., 2016; Voss-Fels et al., 2019; Evans and Lawson, 2020; Gupta et al., 2020; Henry, 2020; Zenda et al., 2021a; Munaweera et al., 2022). However, the large yield gaps still evident in major crops, and our mounting quest to meet human food needs, suggest that there is huge scope for significantly lessening abiotic stress-induced decrease in potential crop yields. Therefore, in view of the foregoing reasons, other avenues for improving crop tolerance to abiotic stresses need to be pursued.

To date, several studies have generated stress tolerant phenotypes by manipulating single traits/genes through the target-gene approach (Umezawa et al., 2006; Reguera et al., 2012; Esmaeili et al., 2022). However, the polygenic nature and complexity of D/+H tolerance suggest that multiple genes or pathways participate in stress response (Shinozaki and Yamaguchi-Shinozaki, 2007; Blum, 2011; Fang and Xiong, 2015; Zhang et al., 2022a), and therefore, conspire against the continued reliant on single-gene targeting to improve such traits; it may not achieve the desire outcome, or may cause inhibition effects on other protein functions or downstream pathways (Zhu et al., 2019; Sharma et al., 2021). Thus, improving plant D/+H tolerance may require deliberate metabolic pathway manipulation (see **Box 1** for definition), through simultaneous targeting of multiple traits/genes within the same or interlinked pathways (Reguera et al., 2012; Zenda et al., 2022). Meanwhile, several candidate metabolic pathways such as *γ-*aminobutyric acid (GABA) biosynthesis (Li et al., 2019; Balfagón et al., 2022), starch biosynthesis (Pinheiro and Chaves, 2011; Hasan et al., 2023), phenylpropanoid biosynthesis (Dong and Lin, 2021) and phytohormonal signalling (Wani et al., 2016) have been implicated in abiotic stress responses. For instance, GABA signalling regulates stomatal opening to enhance plant water use efficiency (WUE) and drought tolerance. In Arabidopsis (*Arabidopsis thaliana*), guard cell GABA synthesis essentially and sufficiently minimizes stomatal opening and transpirational water loss, thereby improving WUE and drought tolerance, through negative regulation of the guard cell tonoplast-embedded anion transporter (Xu et al., 2021a; Xu et al., 2021b). Besides, several metabolites with emerging hormone and antioxidant functions in plants have been identified and implicated in D/+H stress tolerance, including myoinositol, phytomelatonin, trehalose, serotonin, mannose, etc. (Obata et al., 2015; Guo et al., 2018; Itam et al., 2020; Sun et al., 2021; Chen and Arnao, 2022; Raza et al., 2022a). In wheat, for example, a metabolomics study showed that sugars, amino acids, organic acids etc. dominated wheat shoot metabolomic response and enhanced tolerance to drought (Guo et al., 2018). Under prolonged drought stress, osmolytes such as proline, mannose, sucrose, etc., were markedly accumulated, especially in the tolerant genotype JD17. Additionally, drought induced significant alterations in metabolic networks related to tricarboxylic acid cycle, glutamate-mediated proline biosynthesis, glycolysis, shikimate-mediated secondary metabolism and GABA biosynthesis (Guo et al., 2018), suggesting the important role these metabolic pathways play in drought tolerance regulation. Similarly, in soybean (*Glycine max*), myo-inositol and maltose were identified as essential D+H stress biomarkers and were involved in catalase and amino acids biosynthesis pathways (Vital et al., 2022). Additionally, it was observed that under combined D+H stress, network heterogeneity increases whilst integration among metabolic, morphological, and physiological nodes is enhanced (Vital et al., 2022). With metabolite profiles of plant tissues exposed to D/+H revealing a strong relationship between metabolism and grain yield under stress (Obata et al., 2015), such metabolomics studies can provide crucial insights into plant metabolic responses to D/+H stress and reveal novel key potential metabolite biomarkers for engineering D/+H tolerance in cereals (Obata and Fernie, 2012; Michaletti et al., 2018; Vital et al., 2022). However, despite their involvement in diverse abiotic stress response, not much has been achieved in harnessing these candidate metabolic pathways for engineering D/+H stress tolerance in cereals.

In this review, therefore, we discuss how targeted manipulation of specific key metabolic pathways (related to both primary and secondary metabolism), using modern molecular biology tools and approaches such as synthetic biology (Synbio) (see **Box 2**) (Shelake et al., 2022) could help the efficient tailoring of D/+H stress tolerance in cereal crops. In particular, we focus on how carbon metabolism, starch metabolism, GABA biosynthesis, phenylpropanoid biosynthesis and phytohormonal signalling pathways can be deliberately altered to enhance D/+H stress tolerance. First, we briefly highlight the effects of D/+H stress on cereal crops and the corresponding plant responses, before we discuss the deliberate modifications to those key metabolic pathways. We then proffer some perspectives and prospects on metabolic pathway modification for D/+H tolerance, which we hope will invigorate our pursuit to develop climate-smart future crops.

Box 1 **Glossary**

**Biofortification:** an innovative way of increasing crop micronutrient densities through conventional plant breeding, agronomic, or modern biotechnological approaches during the growth of the crop.

**Crop synthetic biology:** an emerging interdisciplinary research field, driven by model design and engineering principles, which involves the construction of novel biological parts, devices and complex systems, or reconstitution of the endemic biological systems for specific useful agronomic and nutritional purposes in crops.

**Differential stress response:** conflicting or contrasting morphological, physiological, biochemical or molecular adjustments (in respect to a specific given trait such as leaf water loss) that plants (cultivars, species, genus, or clades) institute in their pursuit to aptly acclimate or adapt to the imposed stress.

**Metabolic pathway manipulation**: intentional modification of cellular metabolism for improved metabolic productivity for the desired outcomes. It is achieved in different ways, viz., through (i) overexpression of upstream genes encoding rate-limiting enzymes or several key enzymes in the target pathway to increase metabolic flux into that target pathway, (ii) repressing the expression (via knock-out or knock-down) of key enzyme genes in the competitive pathway/s of the branch point/s or the degradation/catabolic pathway of the target product to eliminate intermediates diversion and negative feedback onto the target metabolite, (iii) concomitant expression of multiple target genes within the same pathway, or simultaneous activation of multiple-pathway-involved key (hub) genes from interlinked pathways to increase metabolic flux, and (iv) integration of the above approaches to maximize or optimize the biosynthesis of the target metabolite or molecule (Zhu et al., 2019).

**Metabolic pathway: **a set of molecular interactions between component enzymes/genes and their products that yield to the creation or alteration of some component of the system, underpinning the proper functioning of a biological system. It is connected by intermediates and is linked to other pathways.

**Pathways crosstalk:** interaction between two or more different pathways, which may be metabolism (metabolites biosynthesis and degradation) or signal (stress, growth, or development) transduction-related. A complex network of the converging modules is often created, with the outcome being either synergistic or antagonist depending with the nature of the interaction.

## 2 An overview of the impact of drought or/and heat (D/+H) stress on cereals

The impact of stress on crop plants is dependent upon stress extent and exposure duration, as well as crop species, genotype and growth stage (Gray and Brady, 2016; Fahad et al., 2017). Generally, millets and sorghum can better tolerate D/+H stress than other cereals (Satyavathi et al., 2021; Babele et al., 2022), with certain genotypes exhibiting greater tolerance than others (Azzouz-Olden et al., 2020; Pradhan et al., 2022a). Additionally, the R-stage is more sensitive to D/+H stress than the seedling and vegetative stages (Barnabás et al., 2008; De Storme and Geelen, 2014; Sehgal et al., 2018; Lohani et al., 2020; Chaturvedi et al., 2021; Sinha et al., 2021). Therefore, biotechnological approaches that focus on developing transgenic crops under field or mimicked (close-to-field) conditions and target the reproductive stage will considerably boost chances of creating abiotic stress resilient cultivars (Reguera et al., 2012; Zenda et al., 2022).

Water deficit disrupts numerous cellular and whole-plant functions, exerting negative impacts on plant growth and reproduction (Bray, 1997). Drought stress essentially decreases stomatal conductance, which significantly limit transpiration and CO_2_ assimilation for photosynthesis (Flexas et al., 2004), consequently repressing plant growth and reproduction (Pinheiro and Chaves, 2011). Indisputably, stress disturbs plant cellular homeostasis, hinders key physiological and metabolic processes, which affects overall plant growth (Rivero et al., 2022).

Chiefly, D/+H stress severely affects leaf photosynthesis (Prasad et al., 2008; Pinheiro and Chaves, 2011; Costa et al., 2021), by evoking ROS accumulation in the thylakoid membrane-localized photosystem II (PSII) of the chloroplasts. This causes oxidative stress and damages to photosynthetic pigments and thylakoid membranes, consequently escalating lipid peroxidation, PSII photochemistry inhibition, photosynthesis reactions (electron transfer, ATP synthesis, etc.) depression, programmed cell death, metabolic impairments, and eventually, crop yield reduction (Ramachandra Reddy et al., 2004; Fahad et al., 2017; Hussain et al., 2019; Zhao et al., 2020).

D/+H stress at floral meristem development constricts the overall sink size by decreasing number of florets. Stress inhibits panicle initiation and inflorescence development, resulting in mutilated floral organs, and decreased spikelet number and size (Farooq et al., 2014; Arshad et al., 2017; Begcy and Dresselhaus, 2018; Xu et al., 2021c). Further, D/+H stress causes gametogenesis modification, with the combined stress affecting male reproductive organs more than female reproductive organs (Wang et al., 2019b; Zahra et al., 2021). Pre-anthesis D/+H stress adversely impacts meiosis and ovaries growth, whilst anthesis-stage stress reduces pollen synthesis and transfer, consequently limiting kernel number (Arshad et al., 2017; Qaseem et al., 2019; Choudhary et al., 2022).

Anthesis stage D/+H stress adversely impacts male and female reproductive functions, including pollen germination, pollination, seed set and yield (Barnabás et al., 2008; Alqudah et al., 2011). Combined D+H stress considerably reduce days to anthesis (DTA) and days to maturity (DTM); for example, in bread wheat, DTA and DTM were reduced by 25 and 31%, respectively (Qaseem et al., 2019). In maize, HS alone at pre-anthesis (40/30 °C) and anthesis (36/26 °C) advanced tasselling and pollen shedding duration, reduced the number and viability of pollen shed, and lengthened ASI, consequently reducing final grain yield (Wang et al., 2019b). Meanwhile, anther and pollen development are more prone to stress, which leads to pollination and fertilization failures, and consequently, reduced seed set (Prasad et al., 2006; Djanaguiraman et al., 2020). D/+H stress-induced cytological changes cause drastic effects on several physiological processes, including anther dehiscence, pollen reception, pollen and stigma viability, pollen germination and development, fertilization and seed formation, consequently impacting yield (Farooq et al., 2014; Arshad et al., 2017; Lohani et al., 2020; Chaturvedi et al., 2021; Zahra et al., 2021; Bheemanahalli et al., 2022).

At the seed growth stage, D/+H stress causes abortion of florets, reduced cell expansion and growth, and significant seed size reduction, which all contribute to depressed grain yields and quality in cereals (Sehgal et al., 2018; Costa et al., 2021; Ndlovu et al., 2021; Bheemanahalli et al., 2022). H+D stress arrests cell division and expansion in the central and peripheral endodermis, thereby limiting the breadth and length of the endodermis (Prasad et al., 2008). Subsequently, grain sink potential is considerably reduced; eventually leading to shrivelled grain and decreased mature grain mass (Zahra et al., 2021). At the grain-filling stage, D/+H stress decreases seed weight by quickening the grain-filling duration, consequently diminishing grain yield and quality (Barnabás et al., 2008; Prasad et al., 2008; Barutçular et al., 2016; Djanaguiraman et al., 2020; Ndlovu et al., 2021; Zahra et al., 2021). In rice, for instance, D+H stress at flowering (in Dular cultivar) and grain-filling (in N22 cultivar) caused 73.2 and 77.6% reduction in yields, respectively. Additionally, combined D+H stress at the grain-filling greatly diminished quality, mainly by increasing grain chalkiness in all the three rice cultivars evaluated (Lawas et al., 2018a). Compared to the control, combined D+H stress significantly reduced the 100-seed weight, grain yield plant^-1^ and harvest index (HI) in both maize hybrids evaluated (Hussain et al., 2019).

Meanwhile, studies have shown that different stresses applied individually often impose lesser effects on plant growth and development as compared to the accumulated impact of combined stresses which is detrimental (Zandalinas et al., 2021a; Bheemanahalli et al., 2022). For instance, HS aggravates DS (Hussain et al., 2019; Balfagón et al., 2020; Pradhan et al., 2022a). The combined D+H stress induced more damaging effects on sorghum than the sole (D/H) factors, mainly by increasing canopy temperature considerably (Pradhan et al., 2022a). However, the drought-tolerant genotype Phule Vasudha was less impacted by the exerted stress than the drought-sensitive genotype Phule Revati (Pradhan et al., 2022a). Besides, D, H and D+H triggered oxidative stress, by over-production of ROS and increased MDA contents, which consequently decreased photosynthetic efficiency, nutrients uptake and yield in hybrid maize. The concurrent occurrence of D+H was more severe for maize growth than the individual stresses (Hussain et al., 2019). Taken together, different stress interactions impose varied impacts on plants based on the extent, magnitude and length of the interaction of the involved stress factors (Pandey et al., 2017b), with D+H stress largely exhibiting complementarity that is skewed towards significant negative net impact (yield reduction) (Mittler, 2006). Nonetheless, the impact of combinatorial stress on crops is not automatically accumulative; rather, the result is dependent upon which sole stress factors are involved and how they relate with each other (Pandey et al., 2017b). Therefore, understanding the nature and magnitude of those interactions will be crucial in revealing the actual impact/contribution of each individual and the combinatorial stress on crop plants.

## 3 D/+H stress-induced physiological and molecular responses in cereals

Plant responses to D/+H stress are multi-natured and involve multiple-level adaptations, including morphological (shoot elongation inhibition, root system architecture adjustment, etc.), physiological (stomatal conductance, osmotic adjustment, etc.), biochemical (osmolytes accumulation, antioxidant systems activation, metabolic pathways induction, etc.) and molecular (transcription factor activation, stress-responsive genes up-regulation, etc.) adaptations (Zhao et al., 2020; Vital et al., 2022; Zhang et al., 2022a). Drought and heat tolerance are complex multigenic traits that share some common characteristics with regards to interacting molecular responses and effects on plant growth and development (Georgii et al., 2017; Zandalinas et al., 2018; Jaldhani et al., 2022). For instance both drought and heat inflict oxidative stress damage and dehydration to plant cells (Lipiec et al., 2013; Lamaoui et al., 2018). Additionally, they involve similar components such as stress sensors, protein kinases, phytohormones, transcription factors (TFs), stress-responsive genes and microRNAs (Priya et al., 2019a; Gong et al., 2020; Costa et al., 2021). However, their combination often modifies and yields distinct effects and molecular responses in plants (Zandalinas and Mittler, 2022) which warrant unraveling, especially with regards to multi-factors simultaneously affecting crops in the field.

### 3.1 D/+H-induced physiological responses

In general, plants sense abiotic changes and aptly alter their physiology and metabolism to maximise their productivity at minimum costs (Zhang et al., 2006; Gupta et al., 2020; Moshelion, 2020). Meanwhile, plants tailor their responses to combined stresses, exhibiting some universal and several unique responses (Pandey et al., 2015).

#### 3.1.1 Responses common to individual D and H stresses

Among the common plant physiological responses, a gradual decrease in stomatal conductance and photosynthesis with increasing water stress is characteristic to drought-adapted plants (Ghannoum, 2009). Stomata regulation functions to balance photosynthetic CO_2_ absorption and transpirational water loss (Gosa et al., 2019). In homoiohydric plants, a slight change in vapour pressure deficit (VPD) triggers a rapid stomatal closure to maintain plant water balance (Moshelion, 2020). However, despite its efficiency in water balance maintenance, this passive-hydraulic sensitivity strategy yields less CO_2_ absorption and lower productivity (Moshelion, 2020). On the other hand, plants maintain their water balance via an ABA-driven (chemical-hydraulic) strategy; for instance, guard cells synthesize ABA in response to water-deficit stress (Geiger et al., 2011; Bauer et al., 2013). This mechanism was initially thought to be *de novo* transcription independent, until several microarray analysis studies identified numerous up- or down-regulated genes responsive to exogenous ABA treatment (Leonhardt et al., 2004; Tuteja, 2007; Bauer et al., 2013; McAdam et al., 2016; Kuromori et al., 2018). Especially, *NCED* (*9-CIS-EPOXYCAROTENOID DIOXYGENASE*) gene is up-regulated and mediates extremely rapid *de novo* ABA biosynthesis and stomatal responses to VPD in seed-bearing plants (McAdam et al., 2016). Besides, the site-specific ABA concentrations, for instance in guard cells, depend upon several factors, including biosynthesis, catabolism and inter-tissue or inter-organ transport (Merilo et al., 2015; Merilo et al., 2018). Meanwhile, root-derived ABA potentially govern root water-solute potential dynamics, possibly by modulating aquaporin (AQP) activity, which may essentially facilitate plant adaptation to diverse stress conditions (Kuromori et al., 2018; Kuromori et al., 2022).

Generally, ABA reduces the stomatal sensitivity threshold for VPD such that the stomata are open longer, allowing the plant to be productive for a longer time period. However, this anisohydric mechanism comes at the cost of increased susceptibility to water stress; it is more prone to plant hydraulic failure (Richards, 2000; Moshelion, 2020). Contrarily, isohydric mechanism of VPD response is characterized by rapid stomatal closure and more stable water potential (Tardieu and Simonneau, 1998). Noticeably, crop plants exhibit inter-species, inter-organ, or tissue-specific differences with regards to VPD thresholds and sensitivity to similar environmental stimuli and stress conditions (Moshelion, 2020; Kuromori et al., 2022; Sinha et al., 2022). It has been reasoned that most crop plants are less sensitive to, or synthesize less ABA, in response to stress which reduces their sensitivity to water loss, ultimately elevating their risk of dessication. This has created a productivity-vulnerability dichotomy, whereby more yielding crop cultivars are potentially susceptible to abiotic stresses, due to their rapid growth; greater biomass and sluggish stomatal-closure response (Moshelion, 2020). In view of the foregoing, we must continuously pursue redesigning of anisohydric crop cultivars that could hypothetically sustain higher carbon assimilation rates, or isohydric cultivars that could circumvent CO_2_ starvation under stress conditions. Already, optogenetic manipulation of stomatal kinetics (rate of opening and closing) improved Arabidopsis` CO_2_ assimilation, water use efficiency (WUE) and growth in response to light (Papanatsiou et al., 2019). Notably, the engineered plants produced greater biomass than Wt plants under fluctuating light conditions (Papanatsiou et al., 2019), suggesting that improving stomatal kinetics can potentially enhance WUE, and eventually stress tolerance, without penalty in carbon fixation in crops.

Meanwhile, plants have also evolved various mechanisms to resist D/+H stress, and these mechanisms can be in form of escape, avoidance, tolerance or recovery. Whereas escape involves readjustment of plant phenology to enable completion of a developmental phase or full life cycle prior to the onset of a harmful stress, avoidance involves plants maintaining high tissue water potential under stress (detailed in (Wahid et al., 2007; Aslam et al., 2015; Babele et al., 2022)). Meanwhile, osmotic adjustment, antioxidant systems, phytohormonal regulation, and signal transduction cascades all constitute the tolerance mechanisms (for details, see (Bray, 1997; Wahid et al., 2007; Ndlovu et al., 2021)). Stress recovery involves some plant genotypes surviving the initial stress event and resume their growth once the stress factor is removed; they develop stress memory within their system allowing them to ‘recall’ the stress when it recur and institute apt responses, as aided by epigenetic mechanisms (for details, refer to (Molinier et al., 2006; Chang et al., 2020; Jacques et al., 2021)).

#### 3.1.2 Differential responses unique to combined D+H stress

Literature is replete with studies focusing on plant responses to single stresses, viz., drought (Liang et al., 2018; Danilevskaya et al., 2019; Wang et al., 2019a; Collin et al., 2020; Itam et al., 2020) and heat (Shi et al., 2017; Cai et al., 2020; Mikołajczak et al., 2022). However, in nature, or in the field, plants are often exposed and respond to combined stresses (Mittler, 2006; Lamaoui et al., 2018; Zandalinas et al., 2018; Rivero et al., 2022). Moreover, studies have shown that multi-factor stress produces distinct plant responses that lack direct inference from each sole stress factor responses (Lawas et al., 2018a; Zandalinas et al., 2018). Therefore, it is more useful to investigate the effects of abiotic stress combination and corresponding plant responses (Wani et al., 2016; Zandalinas et al., 2018; Rivero et al., 2022; Sinha et al., 2022; Zandalinas and Mittler, 2022). Thus, here, we shall discuss recently revealed fascinating plant responses to combined D+H stress.

**Figure 1:**
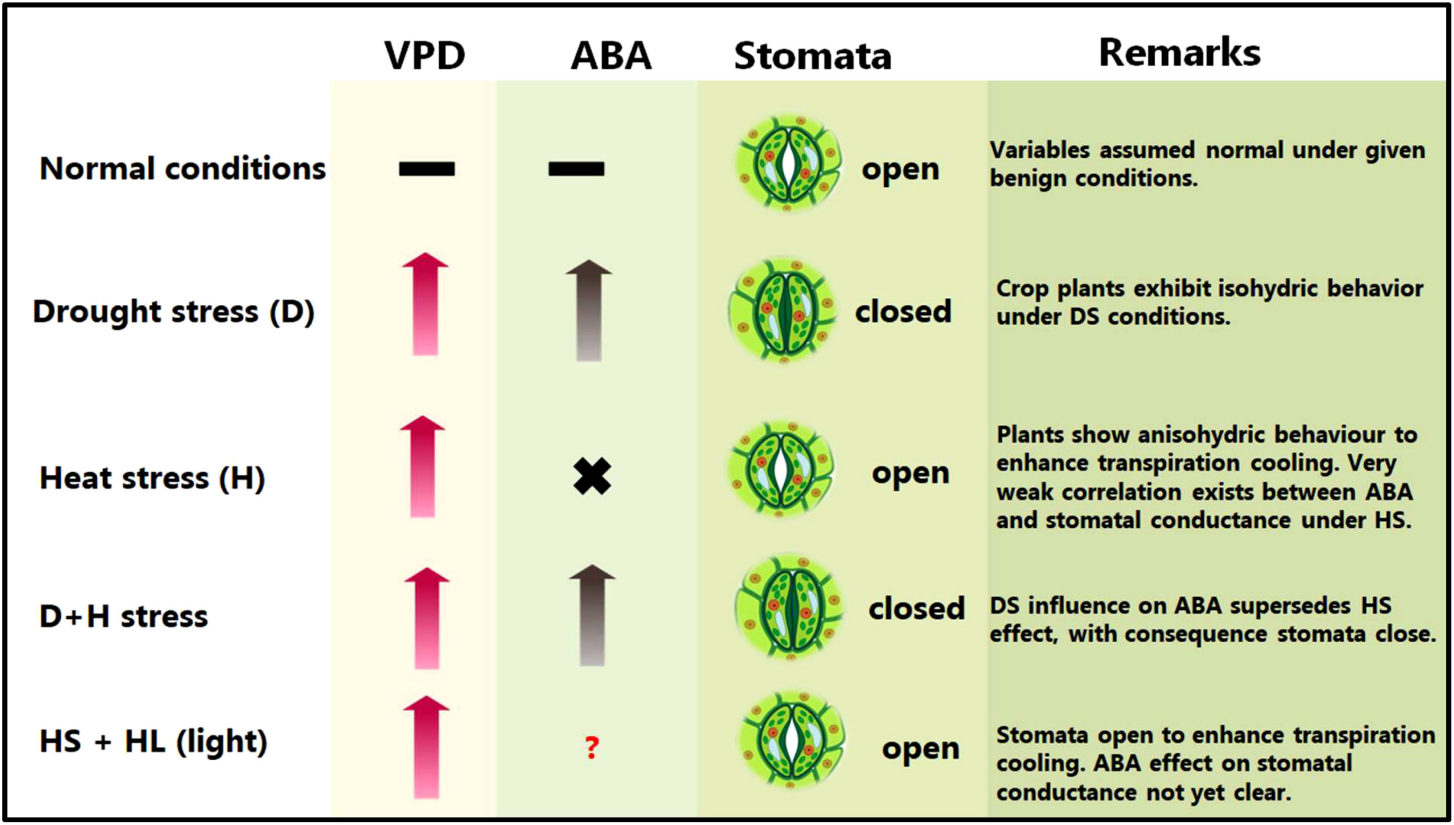
Plant common and differential responses to D/+H stress. VPD, vapour pressure deficit; ABA, abscisic acid. Upward pointing arrows denote increment, hyphens denote optimum or equilibrium (or no significant change) conditions, × implies weak or no correlation, whereas a question mark signifies that the kind of interaction or effects is not yet clear or confirmed.

Abiotic stress combinations induce varied and at times conflicting stomatal regulation behaviours (Rizhsky et al., 2004; Zhang and Sonnewald, 2017; Rivero et al., 2022). For instance, DS causes leaf stomata to close to maintain high plant water balance, whereas HS triggers the leaf stomata to open to enhance leaf transpiration cooling (Zandalinas et al., 2020a; Zandalinas and Mittler, 2022) (**Figure 1**).

During combined D+H stress, leaf stomatal orifice remains shut, implying that DS-driven, rather than HS-driven, stomatal regulation takes precedence (Rizhsky et al., 2004; Zandalinas et al., 2020b). Meanwhile, under H+L stress, HS-driven stomatal regulation supersedes HL (high light)–driven one, which allows stomatal orifice to open under H+L conditions (Balfagón et al., 2019), (**Figure 1**). Intriguingly, despite the established role of ABA in stomatal conductance regulation, no correlation between ABA levels and stomatal closure was observed under D+H conditions (Zandalinas et al., 2016b). These findings may suggest that other regulatory mechanisms, supported by phytohormones such as jasmonic acid (JA) and other processes (eg., ROS stress sensing), may underpin prioritization of certain stomatal responses/behaviours over others under certain stress combinations (Zandalinas et al., 2016a; Balfagón et al., 2019; Balfagón et al., 2020; Sinha et al., 2021; Rivero et al., 2022).

ABA-deficient mutants showed extensive and rapid stomatal closure in response to high VPD, indicative of the passive hydraulic nature of VPD-induced stomatal regulation (Merilo et al., 2018). However, recently, it has emerged that ABA modulates stomatal behaviour by VPD (Li and Liu, 2022). Notably, low VPD impairs stomatal responsiveness (due to lower ABA concentrations). However, DS increases VPD and plants respond by early closure of stomata (due to elevated ABA concentrations) (Li and Liu, 2022). However, plants subjected to HS may not exhibit the same behaviour due to observed lack of correlation between ABA and stomatal conductance under HS conditions (Zandalinas et al., 2016b).

More recently, both leaf and flower stomata have been shown to open under control (non-stress), and both close under DS conditions. Under HS, soybean plants kept both leaf and flower stomata open to maintain transpiration. However, under D+H stress, plants kept the flower stomata open, whilst closing the leaf stomata (Sinha et al., 2022). Authors proposed the opening of flower stomata under HS or combined D+H stress to be a culmination of accelerated ABA catabolism uniquely occurring in flowers on plants grown under those environments. This differential transpiration mechanism helps D+H stress exposed plants to cool their flowers and minimize heat-induced damages onto the reproductive organs (Sinha et al., 2022). In sorghum, contrasting genotypes have exhibited differential leaf canopy cooling in response to D+H stress (Pradhan et al., 2022a). Whereas the drought-tolerant genotype displayed remarkable canopy cool capacity, the drought-sensitive genotype had greater canopy temperature and hotter plant canopy under the imposed stress treatments, suggesting that cooler canopy underpins sorghum adaptation to D+H combination (Pradhan et al., 2022a). The tolerant cultivar might aptly balance moisture conservation and protection from overheating, which helps extend canopy cooling duration until the grain filling stages (Saitou, 1999; Pradhan et al., 2022a). Interestingly, cooler canopies, even under stress, are always associated with higher yields (Zhang et al., 2007; Costa et al., 2021). Previously, wheat sensitivity to D+H stress had been linked to low response of transpiration to high VPD (El Habti et al., 2020), suggesting that maintenance of transpiration and soluble sugars in the grains battling stress are critical for plant D+H stress tolerance.

Moreover, high-resolution dissection of PSII electron transport has revealed differential response to DS and HS in isolation and D+H combined in pearl millet [*Pannisetum glaucum (L.*) R. Br.] (Shanker et al., 2022). The damage to the oxygen evolution complex (OEC) was predominant in heat-stressed, but not in drought-stressed plants. Additionally, OEC damage-induced low exciton absorption flux was evident in HS and H+D stress, causing electron transport congestion in the donor side of PSII (Shanker et al., 2022). These results showed that combined D+H stress was more dominant than the individual stresses on the overall electron transport pathway of the PSII (Shanker et al., 2022).

In view of on-going climate change, combinatorial abiotic stresses and the future of crop productivity, eCO_2_ takes center stage (Lara and Andreo, 2011; Gray and Brady, 2016). Under eCO_2_ conditions, most plants shut stomata, limiting stomatal conductance and water loss (Zhang et al., 2021). Although this may favour plants (especially C4 than C3 species) under DS or D+H conditions by enhancing WUE (Allen et al., 2011; Xu et al., 2013; Ozeki et al., 2022), it may not profit plants acclimating to HS or HS+HL stress that need to maintain stomata open for enhanced cooling (Balfagón et al., 2019; Rivero et al., 2022). Therefore, understanding species differences in eCO_2_ responses in lieu of abiotic stresses will be useful in designing appropriate crop-specific stress tolerance strategies.

### 3.2 D/+H-induced molecular responses

#### 3.2.1 Commonly-shared and convergent stress responses

Plant molecular stress responses encompass stress sensing, signalling, and activation of TFs and stress-responsive genes, as well as post-translational protein modifications (PTMs) and epigenetic alterations [for extensive details, see recent reviews, (Lamers et al., 2020; Zenda et al., 2022; Zhang et al., 2022a)]. Plants alter their signal transduction and metabolic pathways, with ABA and other phytohormones being involved. Once modulated, the signalling pathways elicit TFs activation, ultimately evoking stress-responsive genes and associated metabolic pathways (Zhang and Sonnewald, 2017; Lawas et al., 2018b; Shelake et al., 2022; Zhang et al., 2022a). Notably, antioxidant enzyme encoding genes, conferring osmoprotection, are amplified (Zulfiqar et al., 2019). For instance, OE of *OsRab7* gene confers tolerance to combined H+D stress and improves grain yield in rice through modulation of osmolytes, ROS and stress-responsive genes (*OsSOD-Cu/Zn*, *OsAPX2*, *OsCATA* and *OsCATB*) (El-Esawi and Alayafi, 2019). Besides, overexpressing *ZmHs06* gene improved H+D tolerance in Arabidopsis through enhancing antioxidant capacity (Li et al., 2015a). Altogether, induction of ROS detoxification enzymes has been distinguished as a common response to D+H stress combination in various plant species, suggesting that enhanced antioxidant capacity is associated with plant tolerance to stress combination (Ahanger et al., 2017; Zhang and Sonnewald, 2017; Zandalinas et al., 2018). Several other D+H stress-responsive genes have been identified (Priya et al., 2019a; Esmaeili et al., 2022) (**Table 1**).

**Table 1:** Selected genes useful for D/+H tolerance improvement in cereals using metabolic engineering.

Gene name	Source	Host	Approach	Outcome	Reference
HSFs and HSPs					
*TaHsfA6f*	Wheat	Arabidopsis	OE	Improved sensitivity to ABA, ABA accumulation and tolerance to HS, DS and salinity	(Bi et al., 2020)
*OsHSP18.6*	Rice	Rice	OE	Enhanced antioxidant capacity and improved tolerance to H+D stress	(Wang et al., 2015)
*DREB2A*	Maize	Maize	CE;OE	Enhanced tolerance to D+H stresses	(Qin et al., 2007)
TFs					
*ZmWRKY106*	Maize	Arabidopsis	OE	Improved antioxidant capacity and D+H stress tolerance	(Wang et al., 2018a)
*SNAC3*	Rice	Rice	OE	Enhanced antioxidant capacity, ROS homeostasis and tolerance to H+D stresses.	(Fang et al., 2015)
*Rab7*	Rice	Rice	OE	Improved survival rate, RWC, antioxidant capacity and rice grain yield under D+H stresses.	(El-Esawi and Alayafi, 2019)
*OsMYB55*	Rice	Maize	OE	Enhanced expression of stress-associated genes and improved H+D tolerance	(Casaretto et al., 2016)
*OsWRK11*	Rice	Rice	OE	Reduced water loss and leaf wilting, but increased survival rate and H+D tolerance	(Wu et al., 2009)
*ZmbZIP4*	Maize	Maize	OE	Regulated ABA synthesis and root development, and enhanced stress-responsive genes expression and tolerance to multiple stresses	(Ma et al., 2018)
Stress-responsive genes					
*HVA1*	Wheat	Wheat DHP	OE	Improved ABA sensitivity, reduced oxidative load, and increased D+H tolerance and grain yield	(Samtani et al., 2022)
*TaFER-5B*	Wheat	Wheat and Arabidopsis	OE	Improved tolerance to H+D, oxidative and excess iron stresses.	(Zang et al., 2017)
Protein kinases					
*ZmMAPK1*	Maize	Arabidopsis	OE	Improved ROS scavenging and enhanced D+H stress tolerance	(Wu et al., 2015)
*TaPEPKR2*	Wheat	Wheat and Arabidopsis	OE	Enhanced H+D tolerance in wheat and Arabidopsis plants	(Zang et al., 2018)

HSFs, heat shock factors; HSPs, heat shock proteins; TFs, transcription factors; CDPKs, calcium-dependent protein kinases; OE, overexpression; CE, constitutive expression; Wt, wild type; D+H, combined drought and heat stress; TaPEPKR2, wheat phosphoenolpyruvate carboxylase kinase-related kinase.

Meanwhile, different combinatorial stresses induce considerable gene expression profile readjustments, with HS exerting a dominant effect over osmotic and salinity stresses in relation to global gene expression and relative metabolite abundance changes (Sewelam et al., 2020). Osmotic stress and HS exhibited antagonistic effects on gene expression, with osmotic treatment causing induction of most genes, whilst HS repressed majority of the genes (Sewelam et al., 2020). These divergent stress-induced effects on gene expression may clarify the conflicting physiological responses between D and H stresses discovered earlier on (Rizhsky et al., 2002; Rizhsky et al., 2004; Suzuki et al., 2014; Zhang and Sonnewald, 2017). Moreover, plants enduring combined abiotic stresses (especially where HS is part of the combination) rearrange their transcriptional architecture to repress the induction of most lavish genes (mainly ribosomal and photosynthetic), possibly as a trade-off mechanism to conserve energy and resources for enduring stress (Sewelam et al., 2020). This response, involving down-regulation of redundant proteins to serve energy for battling stress, has been reported previously (Cui et al., 2015; Zenda et al., 2018), suggesting it is a vital abiotic stress acclimation strategy. However, this comes at a cost of reduced productivity. Meanwhile, two categories of usually disregarded genes (the ‘unknown function’ and ‘highly abundant under control conditions’) have been brought to the fore (Sewelam et al., 2020). Since most plant genomes comprise large percentages of ‘unknown function’ genes, there is huge scope for targeting these yet-to-be-characterized genes as novel candidates for engineering abiotic stress tolerance in crops (Luhua et al., 2013). Moreover, the redundant genes may be highly modified or exhibit distinct transcriptional and functional changes under different stress combinations, qualifying them for consideration as potential targets for plant abiotic stress tolerance under such conditions (Shaar-Moshe et al., 2017).

The enormous omics data and gene functional characterization information generated from single stress studies have revealed intriguing convergent stress molecular responses and signalling pathways (Kissoudis et al., 2014; Zandalinas et al., 2020a; Raza et al., 2021; Zandalinas et al., 2021b; Zenda et al., 2021b; Bhardwaj et al., 2022). Exploration of these shared responses, for instance, through meta-analysis, may reveal key candidate genes for combined stress tolerance that can be tested via transgenic approaches. Additionally, understanding the converging signalling pathways, including shared components, can help to pinpoint target metabolic pathways for engineering combined D+H stress tolerance in cereals (Costa et al., 2021). Besides, the functional relevance of other gene types or families (including ion and sugar transporters, protein kinases, TFs, etc.) specifically induced under combined D+H stress conditions can be tested or evaluated (Shelake et al., 2022). In this regard, modern systems biology and Synbio approaches (**Box 2**), to identify core gene regulatory networks, and engineer multiple metabolic pathways and combined stress tolerance, respectively, will be central.

Box 2 **Plant synthetic biology at a nascent stage: can it deliver abiotic stress tolerance in cereals?**

**Synthetic biology** (Synbio) is a fairly new research domain at the intersect of model design and engineering that aims to rationally and systematically construct novel biological systems or modify the existing ones for specific purposes (Serrano, 2007; Liu and Stewart, 2015; Nemhauser and Torii, 2016; Zhu et al., 2021). The engineering principles can be deployed at any level of biological organisation, from molecular to whole-organism (Serrano, 2007), and Synbio has significantly expanded the approaches and tools for conventional biological research (Sargent et al., 2022).

Several modern tools and technologies anchor Synbio, including gene drivers, Golden Gate gene assembly, RNAi, CRISPR-Cas systems, machine learning, artificial gene regulators and promoters, synthetic genetic circuits, biosensors, plastids and metabolic pathways (Kelwick et al., 2014; Braguy and Zurbriggen, 2016; Goold et al., 2018; García-Granados et al., 2019; Lv et al., 2022). Whereas conventional genetic engineering entails manipulation or transfer of individual elements, Synbio can aptly generate complex multigene constructs by simultaneous incorporation or modification of multiple components derived from natural hosts or synthetically synthesized (Goold et al., 2018; Roell and Zurbriggen, 2020). Thus, Synbio enhances the utility of genetic engineering, facilitating for more rapid generation of improved crops harbouring multiple complex traits, which is critical for climate change resilience (Sargent et al., 2022).

Synbio is transforming several disciplines including manufacturing (Köpke, 2022; Scown and Keasling, 2022), food (Lv et al., 2021), and medicine (Xie et al., 2020), and rapidly gaining prominence in agriculture and plant research (Goold et al., 2018; Wurtzel et al., 2019; Steinwand and Ronald, 2020; Llorente et al., 2021). Already, Synbio has been successfully applied to enhance photosynthesis (Głowacka et al., 2018; Batista-Silva et al., 2020; De Souza et al., 2022; Mao et al., 2023), plant disease and pest resistance (Eakteiman et al., 2018; Pixley et al., 2019), and plant nutrition (Roell and Zurbriggen, 2020; Ryu et al., 2020; Yan et al., 2022).

***The key question is: can it deliver abiotic stress tolerance in cereal crops?*** The answer is ‘**yes**’ (Cabello et al., 2014; Yang et al., 2020; Lohani et al., 2022), although several bottlenecks still need to be overcome (Kwok, 2010; Brooks and Alper, 2021; Zhu et al., 2021). Synbio can facilitate D/+H tolerance and yield improvement by enhancing photosynthesis, via re-tuning RuBisCO or other enzymes for better CO_2_ assimilation (Batista-Silva et al., 2020; Qu et al., 2021; Raines, 2022), and integration of multiple genes to enhance photoprotection (De Souza et al., 2022). Additionally, WUE and drought resilience can be improved by introducing novel AQPs (Ermakova et al., 2021), or manipulating ABA biosynthesis via engineering of ABA receptors (Park et al., 2015). Further, it will become more feasible to fine-tune activities of key transcription factors and pleitropic genes to optimize productivity-stress defense trade-offs (Dwivedi et al., 2021; Husaini, 2022). Besides, Synbio can facilitate engineering of genetic circuits able to confer prescribed spatiotemporal gene expression patterns. For instance, root development can be redesigned by quantitatively controlling lateral root density (Brophy et al., 2022). Remarkably, Synbio can essentially facilitate trait/gene stacking or metabolic pathways integration, which enables creation of complex and effective crop tolerance to certain stress combinations.

One of the key challenges in Synbio is how to rationally create new genetic circuits capable of achieving predictable functions in a diverse range of conditions (García-Granados et al., 2019; da Fonseca-Pereira et al., 2022). Another bottleneck relates to the limited transferability of Synbio platforms and products to ‘outside-the-lab’ resource-limited and off-the-grid settings, since they lack long-term storage capabilities, flexibility and amenability to limited equipment and human intervention (Brooks and Alper, 2021). Besides, technical bottlenecks related to the identification of precise gene/s for targeted functions still persist, especially when dealing with multigenic functions (Sargent et al., 2022). Moreover, most Synbio-based techniques are not amenable to cereal species, which already possess inherent tissue-culture-transformation-incompatibilities due to recalcitrance (Silva et al., 2022). Further, several significant ethical concerns comes to the fore, including potential health hazards and ecological consequences linked to genetically modified organisms (GMOs) (Wang and Zhang, 2019). Biosafety regulatory issues - costs and complexity of compliance with biosafety regulatory requirements, as well as social acceptance limit R&D and deployment of GMO products (Pixley et al., 2019; Wurtzel et al., 2019; Sargent et al., 2022). Nevertheless, the expansion of Synbio field opens up new possibilities for abiotic stress tolerance improvement in crops and future climate-smart agriculture.

#### 3.2.2 Transcriptional regulation of D/+H stress responses

Transcription factors (TFs) are key transcriptional regulators of drought (Nakashima et al., 2014; Singh and Laxmi, 2015; Joshi et al., 2016; Manna et al., 2021) and heat (Guo et al., 2016; Ohama et al., 2017; Zhao et al., 2020; Haider et al., 2022; Zenda et al., 2022) stress responses in plants. TFs link signaling pathways with downstream gene regulation; once modulated by these signaling pathways, TFs directly or indirectly interact with *cis*-acting elements to regulate the transcriptional programs of their target genes (Weidemüller et al., 2021). Both ABA-dependent and ABA-independent signal transduction pathways underpin transcriptional responses to drought (Yamaguchi-Shinozaki and Shinozaki, 2006). Several TFs such as *ABSCISIC ACID-RESPONSIVE ELEMENT BINDING PROTEIN1* (*AREB1*), *DEHYDRATION-RESPONSIVE ELEMENT BINDING PROTEIN 2A/2B* (*DREB2A/2B*)*, MYC*/*MYB*, *RD22BP1*, etc., mediate the ABA-responsive mechanism, via interaction with their corresponding cis-acting elements such as *ABRE*, *DRE*/*CRT* (DRE/C-repeat sequence), *MYCRS*/*MYBRS*, respectively (for details, see (Tuteja, 2007)). These upstream TFs modulate cis-regulatory elements (CREs), such as *DRE/CRT* (A/GCCGAC), *ABRE* (PyACGTGGC), MYCRS (MYC recognition sequence, CANNTG) and MYBRS (MYB recognition sequence, C/TAACNA/G), harbored in the promoters of stress-induced genes (Fujita et al., 2005; Yamaguchi-Shinozaki and Shinozaki, 2006; Fujita et al., 2013). The canonical ABA-SnRK2s-PYR/PYL/RCAR-PP2C-ABF/AREB signaling module drive the ABA-dependent pathway (Umezawa et al., 2009; Fujita et al., 2013), whereby ABA accumulation triggers class III SnRK2 (SUCROSE NON-FERMENTING-1 RELATED PROTEIN KINASE 2) protein kinases induction, via the PYR/PYL/RCAR-PP2C [PYRABACTIN RESISTANCE1/PYR1-LIKE/REGULATORY COMPONENTS OF ABA RECEPTOR - PROTEIN PHOSPHATASE 2C] complex, and *AREB1*, *AREB2*, *ABF3* (ABRE binding factor 3), and *ABF*1 are phosphorylated under drought stress conditions to regulate the expression of downstream target genes (for details, see (Fujita et al., 2009; Yoshida et al., 2010; Fujita et al., 2013; Hsu et al., 2021)). *AREB* induce the expression of *RD29B* gene (Uno et al., 2000), whereas MYC/MYB TFs, *RD22BP1* and *AtMYB2* bind MYCRS and MYBRS, respectively, to induct *RD22* gene (Uno et al., 2000; Tuteja, 2007). The activation of these genes relies on the build-up of endogenous ABA levels, suggesting their later-stage involvement in drought stress response (Tuteja, 2007). Meanwhile, *DREB2A/2B* and other ABA-reliant drought-responsive TFs trans-induct several stress-responsive genes (reviewed/listed in (Singh and Laxmi, 2015; Todaka et al., 2015; Joshi et al., 2016); **Table 1**). For instance, *ZmDREB2A* overexpressed in Arabidopsis improved transgenic plants` H+D stress tolerance, through influencing LEA (late embryogenesis abundant), heat shock, and detoxification encoding genes (Qin et al., 2007). Overexpressed *ZmHsf06* enhanced H+D tolerance in transgenic Arabidopsis, possibly by increasing SOD, POD, CAT activities and ROS homeostasis (Li et al., 2015a). Other TF families such as WRKY and MYB also participate in ABA-dependent pathway. For example, overexpreed *TaWRKY1* and *TaWRKY33* confer D/+ H tolerance in transgenic Arabidopsis, by activating several stress-responsive genes (He et al., 2016). *TaWRKY1* exhibits slight up-regulated response to HS and ABA, whereas *TaWRKY33* shows high responses to HS, ABA, and MeJA (jasmonic acid methylester) (He et al., 2016).

The ABA-independent DS response regulation mechanism involves DREB and other TFs such as NAC [NAM, ATAF, and CUC], WRKY, MYB/MYC, NF-Y (nuclear factor-Y), etc., in modulating several drought-responsive genes (Fujita et al., 2013; Singh and Laxmi, 2015). For instance, *OsNAC016* regulates crosslinking of BR-mediated plant architecture (positive influence) and ABA-mediated drought tolerance (negative influence) in rice, by interacting with GSK2 and SAPK8 kinases via PTMs (Wu et al., 2022). However, expression of *OsWRKY5* is decreased by DS, ABA, NaCL, mannitol treatments, suggesting that inactivation of *OsWRKY5* improves rice DS tolerance (Lim et al., 2022). Meanwhile, *ZmNF-YC12* is highly induced by drought and rewatering treatments, and modulates drought tolerance and recovery ability in maize, by inducing genes related to improved photosynthesis and antioxidant capacities (Cao et al., 2023). Similarly, overexpressed *ZmNF-YA1* and *ZmNF-YB16* modulated maize plant growth and drought tolerance, via induction of genes related to root development, photosynthesis and antioxidant capacity (Yang et al., 2022). Besides, these TFs cross-talk with each other or with phytohormones such as brassinosteroids (BRs) for efficient regulation of stress response ((Nakashima et al., 2014; Singh and Laxmi, 2015; Jogawat et al., 2021); also discussed here in detail later under ‘*Phytohormone biosynthesis and signalling pathway*s’ section). Therefore, identifying and manipulating those key/hub stress-responsive TFs cross-linking several pathways (for instance, through Synbio) offers much better prospects of improving D/+H stress tolerance than attending to each functional gene individually (Joshi et al., 2016; Tenorio Berrío et al., 2022).

HEAT SHOCK FACTOR A1 (HSFA1) centrally activates transcription and HS response, by stimulating immediate induction of other HS-responsive TFs such as DREB2A, HSFA7, HSFBs, etc. (Ohama et al., 2017). HSFA1 also transactivates other HSFs (DREB2A, HSFA2, HSFA3, HSFA7, etc., to trigger the expression of other HS-inducible genes (reviewed in (Guo et al., 2016; Haider et al., 2022)). This is achieved through HSFA1 crosslinking with HSP70 and HSP90 under HS (Jacob et al., 2017). For instance, *HSFA1* directly targets *DREB2*, which regulates *HSFA3* by creating a coactivator complex with *NF-YA2*, *NF-YB3* and *DPB3-1/NF-YC10*, whose affinity to bind to HSFA3 promoters induct HSFA3 expression (Schramm et al., 2008). More importantly, DREB2A integrates HS and DS responses by triggering the corresponding sets of stress-responsive genes, including LEA proteins and HSPs (Guo et al., 2016; Zhao et al., 2020). These HSPs (HSP70, HSP90, HSP100, etc.) and sHSPs are actively recruited to regulate protein homeostasis, by repairing or replacing HS-damaged proteins (Al-Whaibi, 2011); thus, their molecular chaperone function positively modulates D/+H tolerance ((Jacob et al., 2017); **Table 1**). For instance, *OsHSP50.2*, an HSP90 family gene, overexpressed in rice, promoted DS tolerance, possibly through modulating ROS homeostasis and osmotic adjustment (Xiang et al., 2018). Meanwhile, upon HS, cytosolic *HSP70-3* interacts with plasma membrane-embedded PLDδ (phospholipase Dδ) to stabilize cortical microtubules, facilitate phospholipid metabolism, and enhance HS tolerance in Arabidopsis (Song et al., 2020). Other TF families that modulate HS-responsive genes include WRKY, NAC, MYB, AP2/EREBP, bZIP, etc. (Wang et al., 2016; Zhao et al., 2020). For example, *OsWRKY11* constitutively expressed under the control of *HSP101* promoter improved H+D tolerance in rice (Wu et al., 2009). Of note, we have extensively detailed the molecular mechanisms of HS response in cereals in our more recent review (Zenda et al., 2022); therefore, we refer readers to that article. Taken together, regulation of stress-responsive genes by TFs, crosslinking with phytohormonal and stress signaling pathways underlie D+/H stress responses in plants; increased understanding of these mechanisms helps to reveal key hub TFs, genes or candidate pathways for engineering D/+H tolerance in major crops, including cereals.

#### 3.2.3 Epigenetic regulation and non-coding RNAs-mediated modulation of D/+ H stress response

Epigenetic regulation mechanisms (eg., histone modification, DNA methylation, chromatin remodelling, etc.) (Banerjee and Roychoudhury, 2017; Begcy and Dresselhaus, 2018; Liu et al., 2022; Singh and Prasad, 2022), small RNAs (sRNAs, 18-30 nucleotides (nt) long) (Zhang, 2015; Banerjee et al., 2017; Wani et al., 2020; Zhou et al., 2020) and long noncoding RNAs (lncRNAs, > 200 nt) (Nejat and Mantri, 2018; Yu et al., 2019; Chang et al., 2020; Jha et al., 2020) have emerged as essential modulators of various plant abiotic stress responses (Zhao et al., 2020; Miryeganeh, 2021; Zenda et al., 2021a; Zhang et al., 2022a). Histone modification and DNA methylation regulate gene expression responses to abiotic stresses [see (Liu et al., 2022)]. Histone acetyltransferase (HATs) promote enhanced gene expression through acetylation/relaxation of chromatin from histones (Ueda and Seki, 2020) whilst DNA methyltransferases underpin transcriptional repression of transposable elements (Zhang, 2015; Begcy and Dresselhaus, 2018). Recently, histone acetyltransferase *TaHAG1* has been shown to interact with *TaNACL* to promote HS tolerance by maintaining photosynthetic stability in wheat (Lin et al., 2022).

Non-coding RNAs (ncRNAs), lacking obvious protein coding capacity, and comprising sRNAs, lncRNAs, circular RNAs (circRNAs), etc. (Yu et al., 2019; Bhogireddy et al., 2021), crucially regulate plant growth, development and stress response processes, by modulating transcriptional and post-transcriptional expression of target genes, and fine-tuning growth-stress defense trade-offs (Wang et al., 2017; Zhang et al., 2022b). These ncRNAs interact with their targets to create complex gene regulatory networks that orchestrate metabolic reprogramming essential for D/+H tolerance (Bhogireddy et al., 2021; Gelaw and Sanan-Mishra, 2021). sRNAs, especially microRNAs (miRNAs), target TFs or sequester mRNA (messenger RNA) cleavage sites to control gene activation or post-transcriptional translation inhibition (Zhang, 2015; Li et al., 2019). Several plant stress-responsive *miRNAs* have so far been discovered (Li et al., 2019; Zhou et al., 2020; Zahra et al., 2021). For instance, miR*398* actively participates in HS response regulation, as a direct target for HSFA1 (Ohama et al., 2017), and is induced by HS (Guan et al., 2013). miRNA398 chiefly target ROS-scavenging genes, viz., Cu/Zn superoxide dismutases (cytosolic *CSD1*, and chloroplastic *CSD2*), *CCS1* (a Cu chaperone for SOD), et. (Sunkar et al., 2006). Rapid induction of *miRNA398* under HS reduces transcripts of *CSD1*, *CSD2* and *CCS1* (Guan et al., 2013). On the other hand, increased transcript levels of *CSD1* and *CSD2* down-regulates *miR398* transcription under oxidative stress, with this feedback loop being critical for *CSD1* and *CSD2* mRNA accumulation post-transcriptionally and oxidative stress tolerance (Sunkar et al., 2006). Overall, this reveals the importance of *miR398*-*CSD*/*CCS*-*HSF* pathway in plant HS response (Zhao et al., 2016). Meanwhile, the induction of *miR156* under HS post-transcriptionally down-regulates *SQUAMOSA-PROMOTER BINDING-LIKE* (*SPL*) genes in Arabidopsis, which is vital for HS memory (Stief et al., 2014). The created *miRNA156-SPL* module, thus, critically mediates HS memory and tolerance (Stief et al., 2014; Zhao et al., 2016).

Long non-coding RNAs (lncRNAs) also actively participate in D+/H stress response regulation (Chen et al., 2020; Jha et al., 2020). They underpin several regulatory mechanisms, including acting as target mimics (decoy RNAs) for miRNAs to thwart interactions between miRNAs and their authentic targets, serving as sRNA precursors to generate sRNAs (miRNAs, siRNAs, etc.), antisense lncRNAs interacting with sense mRNAs to form natural antisense transcripts (NATs) which regulate gene expression, lncRNA-meditated chromatin modifications (eg., lncR2Epi pathway), and RNA-directed DNA methylation (RdDM) pathway, all of which orchestrate stress response in one way or the other (excellently detailed in (Wang et al., 2017)). For example, the lncRNA *DANA2* has been recently shown to recruit an AP2/ERF transcription factor ERF84 to evoke *Jumonji 29* (*JMJ29*)-mediated histone demethylation and positively regulate drought tolerance in Arabidopsis (Zhang et al., 2023). In rice, 98 drought-responsive lncRNAs modulated several drought-responsive regulatory genes involved in different metabolic processes (Chung et al., 2016). Meanwhile, 231 heat-responsive lncRNAs have been identified and characterized in two rice cultivars contrasting in heat tolerance (Zhang et al., 2022c). Notably, as mediated by *osa-miR1439*, some heat-responsive lncRNAs co-interacted with protein coding genes (eg., *TCONS_00001878* with *Os01g0104900*, *TCONS_00030558* with *Os01g0196800*, etc.*)* to form ceRNA (competing endogenous RNA) pairs in the heat-sensitive cultivar SYD2 (Zhang et al., 2022c). Previously, *osa-miR1439* exhibited induced expression under high temperature, revealing that *osa-miR1439* possess a specific function in HS-response regulation (Mangrauthia et al., 2017). Equally, lncRNAs potentially modulate HS responses via a ceRNA mode involving lncRNA-*osa-miR1439*-regulatory gene circuits (Chen et al., 2020; Zhang et al., 2022c). In maize, 53 249 (including 259 known and 52 990 unknown) heat-responsive lncRNAs were identified, among which 993 lncRNAs showed significant differential expression under HS (Hu et al., 2022). The *cis*- and *trans*- regulation mechanisms involving these differentially expressed lncRNAs shared 953 common gene targets. Several important biological processes and stress response-related pathways, including photosynthesis, hormone signal transduction, etc. were enriched in these shared gene targets, revealing their crucial involvement in HS response (Hu et al., 2022).

Meanwhile, circRNAs have been suggested to act as miRNA sponges under heat and drought stress conditions, in Arabidopsis and wheat, respectively (Litholdo and da Fonseca, 2018). Besides, endogenous RNAs (miRNAs, lncRNAs, circRNAs, etc.) compete with miRNA recognition elements (MREs) for miRNA binding sites and, thus, regulate each other in the process; the dynamic balance of endogenous RNAs is therefore critical in regulating plant cellular homeostasis under stress conditions (Zhou et al., 2020). Here, we underscore that systemic uncovering and analysis of key stress-responsive epigenetic marks, sRNAs and lncRNAs and their target genes could facilitate their endogenous modification and tailoring of abiotic stress tolerance in cereals (Banerjee et al., 2017; Sihag et al., 2021; Ali et al., 2022; Liu et al., 2022). Moreover, accruing a repertoire of novel abiotic stress-associated sRNAs and lncRNAs from diverse clades facilitates rigorous and dynamic stress resilience in those rationally created varieties (Zhang, 2015; Zenda et al., 2022).

## 4 Key pathways targeted for manipulation

In this section, we will discuss the key primary metabolism- and secondary metabolism-related pathways that can be modified using modem biotechnological approaches to enhance cereal crops growth and yield under D/+H stress conditions.

### 4.1 Carbon metabolism

Targeting improved photosynthesis remains a topical strategy for enhancing crop productivity and abiotic stress tolerance (Simkin et al., 2015; Nowicka et al., 2018; Furbank et al., 2020; López-Calcagno et al., 2020; Zhu et al., 2022b). For decades, RuBisCO (ribulose-1,5-bisphosphate carboxylase-oxygenase - an enzyme that catalyses the first rate-limiting step in CO_2_ fixation) has been the main engineering focus for enhancing plant photosynthesis efficiency, through its expression modification in transgenic plants (reviewed in (Orr et al., 2017; Sharwood, 2017; Roell and Zurbriggen, 2020)). However, several new targets have emerged. For instance, considering the fundamental role Calvin–Benson–Bassham (CBB) cycle plays in primary carbon metabolism, modifying the expression of other CBB cycle-involved enzymes (eg. ribulose-1,5-bisphosphate; sedoheptulose-1,7-bisphosphatase; chloroplastic fructose-1,6-bisphosphatases, etc.) can also improve photosynthetic capacity and growth (Raines, 2022). Especially, retuning RuBP regeneration, via simultaneous incorporation of proteins that function outside of the CBB cycle, can significantly improve photosynthesis and plant growth over single gene manipulations (Simkin et al., 2015; Simkin et al., 2019; Raines, 2022). Besides, increased expression of brassinole resistant 1 (BZR1) TF amplified the expression of a set of CBB cycle genes (*RCA1*, *FBA1*, *PGK1* and *FBP5*) and improved photosynthetic capacity, revealing that concurrent OE of these multiple proteins can invigorate the CBB cycle (Yin et al., 2021; Raines, 2022).

Crop yield is determined by photosynthetically active radiation (PAR) availability, PAR capture efficiency, light energy conversion (into biomass) and harvest index (Long et al., 2006; Simkin et al., 2019; Roell and Zurbriggen, 2020). Whilst all other determinants have reached their potential maxima, energy conversion is still < 40% of its theoretical potential (due to photorespiration losses), representing, therefore, a potential engineering target (Long et al., 2015; Simkin et al., 2015; Slattery and Ort, 2015). Key strategies for improving plant carbon metabolism include boosting carboxylation efficiency (via repurposing efficient CO_2_-concentrating mechanisms, eg., C4 photosynthesis, cyanobacterial carboxysomes or pyrenoids) (Nowicka et al., 2018; Adler et al., 2022; Pradhan et al., 2022b), minimizing photorespiratory and respiratory CO_2_ losses (for instance, via engineering of chloroplastic photorespiratory bypasses) (Roell and Zurbriggen, 2020; da Fonseca-Pereira et al., 2022), developing synthetic and more efficient CO_2_ fixation routes (such as the construction, *in vitro*, of crotonyl–coenzyme A (CoA)/ethylmalonyl-CoA/hydroxybutyryl-CoA (CETCH) cycle) as the CBB cycle surrogates (Long et al., 2015; Kubis and Bar-Even, 2019; South et al., 2019), creating more efficient photoprotection systems to minimize heat dissipation (De Souza et al., 2022), RuBisCO reengineering for enhanced catalytic rate and greater specificity for CO_2_ (Orr et al., 2017; Batista-Silva et al., 2020; Iqbal et al., 2021a; Mao et al., 2023), and development of synthetic (artificial) systems that tolerate high light conditions (Yu et al., 2018; Zhu et al., 2020; Raines, 2022; Zhu et al., 2022b). Fortunately, the availability of versatile tools such as Synbioand nanomaterials is facilitating targeted manipulation of these photosynthesis aspects for improved abiotic stress resilience and enhanced yield (Raines, 2022).

High complexity and crosstalk of photosynthesis and abiotic stress response pathways (which often impact multiple pathways) dictates that traits aimed at improving photosynthetic efficiency and resilience to combined stresses call for targeted multiple-gene or/and novel reaction pathways integration (Nowicka et al., 2018; Lata and Shivhare, 2021; Sargent et al., 2022). For instance, for a highly efficient photosystem, tissue-specific promoters can be used to precisely regulate specific spatio-temporal expression of genes encoding photosystem components, such as *psaAB* and *psbA* (encoding the reaction centre apoproteins of PS I, and the D1 protein of PS II, respectively) (Pfannschmidt et al., 1999; Zhu et al., 2020). Synbio tools such as CRISPR-Cas9 [clustered regularly interspaced palindromic repeats (CRISPR)-Cas9 (CRISPR-associated protein 9)] can now competently perform transference of lengthy gene constructs with customized expression profiles or facilitate fine-tuning of gene expression levels (Kubis and Bar-Even, 2019; Sargent et al., 2022). Moreover, considering the intricate nature of the photosynthesis system, it now more plausible to exchange complete photosynthetic multi-protein complexes (instead of individual components) between different species (Batista-Silva et al., 2020). Further, single-cell transcriptomics and stereomics are now enabling identification of novel gene promoters conferring spatiotemporal, phonological or environment specificity (Xia et al., 2022).

Meanwhile, high-throughput plant phenotyping platforms (HT3Ps) integrated with genomic-wide association studies (GWAS) are facilitating the discovery and characterization of novel traits/genes underpinning photosynthetic efficiency (Zhu et al., 2020; Araus et al., 2022). Besides, multi-scale systems modelling of photosynthesis enables not only dissection of mechanisms regulating the competence of certain photosynthetic proteins or complexes, but also the custom designing of optimized photosynthesis machineries with enhanced efficiency under diverse stress environments (Xiao and Zhu, 2017). Taken collectively, new technologies now offer unprecedented opportunities to design completely new photosynthesis systems tailored for combined abiotic stress conditions (Zhu et al., 2022b).

Other potential ways to improve crop biomass production encompass engineering of specific proteins (such as ion transporters) or phytohormones (Nowicka et al., 2018), and enhancing antioxidant capacities of plants under field and combined stress conditions (Zhu et al., 2020). However, it is worth noting that precise photosynthetic limitations vary between species, for instance, the rates of stomatal conductance, canopy structure, etc. (Kromdijk and McCormick, 2022); therefore, photosynthesis engineering strategies need to be tailored to each species. Besides, given that growth-defense trade-off is a critical survival mechanism in plants (Dwivedi et al., 2021), novel genetic and Synbio tools will facilitate rewiring of plant fitness programs and promote/optimise concomitant plant biomass production and stress defense. Furthermore, tailoring of root traits and HSPs (Hu and Xiong, 2014; Reddy et al., 2016; Lawas et al., 2018b; Rahman et al., 2022), when integrated with photosynthetic enhancements, could enhance crop D+H stress tolerance and yield (López-Calcagno et al., 2020; Sargent et al., 2022).

Thus, considering that several attempts to modify single core traits/components has generally yielded undesired effects (Sweetlove et al., 2017), largely due to the interactive nature of most metabolic pathways (Batista-Silva et al., 2020; da Fonseca-Pereira et al., 2022), we amplify the view that targeted manipulation of multicomponent traits and/or metabolic pathways (preferably concomitantly) offers great promise for managing such complexity, enhance overall plant system performance, improve combined abiotic stress resilience and productivity (Sargent et al., 2022; Shelake et al., 2022). Synbio integrated with other modern tools, including systems biology, computational and multi-omics approaches will drive this pursuit (Perez de Souza et al., 2022; Zhan et al., 2022) (**Box 2**).

### 4.2 Starch metabolism

Uncovering of the plasticity of starch metabolism under abiotic stress conditions supports that starch metabolism alterations crucially regulate plant responses to abiotic stresses such as salinity, drought and heat (MacNeill et al., 2017; Thalmann and Santelia, 2017). For instance, under stress and constrained photosynthesis conditions, starch reserves are remobilized to provide energy, sugars and derived metabolites, subsequently helping plants to alleviate stress (Thalmann and Santelia, 2017; Hasan et al., 2023). The released sugars, besides providing the osmoprotection function, may act as primary stress signal transducers, and crosstalk with phytohormones such as ABA, SA, JA, etc. This fortifies plant responses to the stress (Rosa et al., 2009; Jogawat et al., 2021; Saddhe et al., 2021). Further, soluble sugar levels modulate gene expressions and enzyme activities in both sugar exporting and sugar importing tissues (Roitsch, 1999; Gupta and Kaur, 2005; Pinheiro and Chaves, 2011), thereby optimizing synthesis and utilization of carbon and energy resources (Rosa et al., 2009; Yoon et al., 2021). For instance, soluble sugars and metabolites levels were considerably increased under H+D stress in the floral organs of the tolerant rice genotype N22 (Li et al., 2015b). Nine key metabolites, mostly TCA cycle- and sugar metabolism-related (sucrose, myo-inositol, succinate, etc.), were suggested to confer tolerance to H+D stress, among which six had greater accumulation in N22 genotype. More strikingly, sucrose level was significantly decreased in the susceptible genotype, suggesting that sugar starvation contributes to reproductive failure under H+D stress (Li et al., 2015b). Besides, the resistant cultivar N22 showed greater expression of genes encoding sugar transporter (*MST8*) and cell-wall invertase (*INV4*) under H+D stress, signifying these genes` key role in combined H+D stress tolerance (Li et al., 2015b).

**Figure 2:**
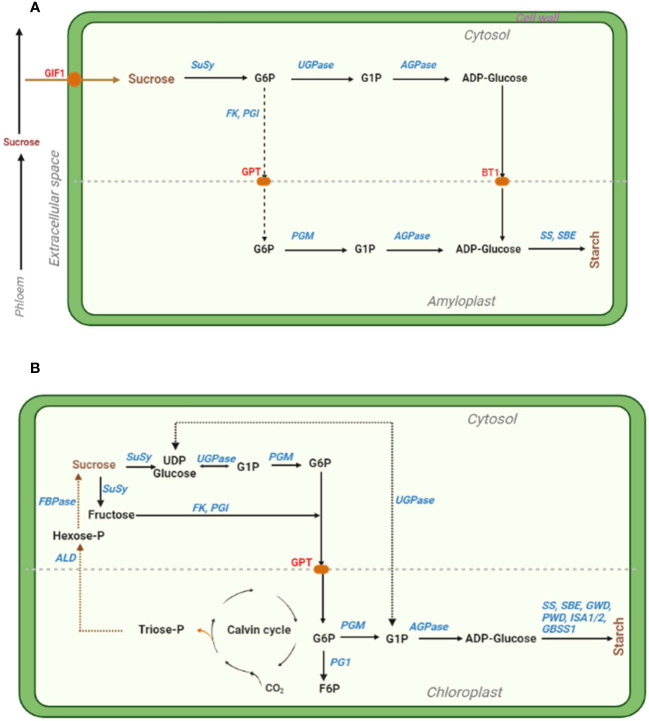
Simplified illustration of the starch biosynthesis pathways in cereal endosperm **(A)** and photosynthetic leaf **(B)** cells, with key enzymes that can be targeted for manipulation shown in blue. **(A)**. The cytosolic and amyloplastic compartments are demarcated by the light gray longitudinal dashed line. Sucrose is converted into starch (mainly amylose and amylopectin) through a series of enzymatic steps, involving glucose 6-phosphate (G6P), glucose 1-phosphate (G1P) and adenosine diphosphate glucose (ADP-Glucose). The enzymes are abbreviated as follows: SuSy, sucrose synthase; UGPase, UDPglucose pyrophosphorylase; FK, fructokinase; PGI, phosphoglucose isomerase; PGM, phosphoglucomutase; AGPase, ADPglucose pyrophosphorylase; SS, starch synthase; SBE, starch-branching enzyme. The red pods identified in red font denote sucrose transporters as follows: *GIF1*, *GRAIN INCOMPLETE FILLING 1*; GPT, glucose 6-phosphate transporter; and BT1, *BRITTLE1* (an ADPglucose/ADP antiporter transporter). The dotted arrows signify a series of steps of the fructose-mediated pathway. Adopted from (Comparot-Moss and Denyer, 2009; MacNeill et al., 2017; Huang et al., 2021). **(B)**. Sucrose is converted into starch through a series of enzymatic steps, involving Uridine diphosphate glucose (UDP glucose), G1P, G6P, and ADP-Glucose. Meanwhile, G6P in the chloroplast can also be converted into fructose-6-phosphate (F6P). Triose-phosphate (Triose-P) generated from the Calvin cycle is converted through a series of events into sucrose and stored in sinks. Other enzymes GWD, glucan water dikinase; PWD, phosphoglucan water dikinase; ISA1/2, isoamylase 1/2; GBSS1, granule-bound starch synthase 1; ALD, aldolase; FBPase, fructose-1,6-bisphosphatase. Adopted from (Comparot-Moss and Denyer, 2009; Geigenberger, 2011; Hasan et al., 2023).

Several enzymes catalyse starch biosynthesis, including the cytosolic ADP-glucose pyrophosphorylase (AGPase), UDP-invertase, sucrose synthase (SuSy), etc. and plastidial starch synthase, starch-branching enzyme, etc. [reviewed in (Comparot-Moss and Denyer, 2009; Cho and Kang, 2020; Huang et al., 2021)] (**Figure 2**). Especially, altering AGPase, one of the main enzymes catalyzing the rate-limiting and first committed key enzymatic step of starch biosynthesis (Comparot-Moss and Denyer, 2009), can enhance the regulation of starch synthesis and distribution under combined D+H stress conditions (Saripalli and Gupta, 2015). Therefore, thermotolerant variants of AGPase can be harnessed (eg. via overexpression) to develop HS tolerant wheat (Kang et al., 2013) and maize (Li et al., 2011) cultivars with enhanced starch biosynthesis and higher grain weight. Thus, altering AGPase to enhance leaf starch biosynthesis and during grain filling (using seed-specific promoters) potentially improves grain yield and abiotic stress tolerance in cereals (Saripalli and Gupta, 2015). Further, boosting ADPglucose transportation into amyloplast and modification of other enzymes involved in photoassimilate partitioning into storage organs has the potential to increase plant productivity and stress tolerance (Tuncel and Okita, 2013). For example, engineering a heat-stable plastidial 6-phosphogluconate dehydrogenase (6PGDH) enhanced grain yield in heat-stressed transgenic maize (Ribeiro et al., 2020). To improve heat stability of the amyloplast-localized and heat-labile, but critical grain-starch-accumulation-involved enzyme PGD3, authors used endosperm-specific promoters to target/import 6PGDH into endosperm amyloplasts by fusing the *Waxy1* chloroplast. Consequently, *WPGD1* and *WPGD2* transgenes showed improved 6PGDH activity and heat stability *in vitro*, complemented the *pgd3-*defective kernel phenotype, and reduced high night temperature-induced grain yield loss via increased kernel number (Ribeiro et al., 2020).

Different abiotic stresses induce differential source-sink dynamics that evoke differential expression of various carbohydrate metabolism-related proteins/genes (starch-biosynthesis and starch-degrading or sucrose metabolism enzymes) (Roitsch, 1999; Rosa et al., 2009; Pinheiro and Chaves, 2011; Tuncel and Okita, 2013). In general, storage proteins (eg. sporamin) are induced, whereas sucrose metabolism-related proteins (eg. α-amylase and sucrose synthase) are repressed under abiotic stresses (see (Gupta and Kaur, 2005; Rosa et al., 2009)), with sucrose-specific signalling pathways mainly repressing *ATB2 bZIP* TFs (Wiese et al., 2005; Yoon et al., 2021). Meanwhile, different combinations of starch-degrading enzymes accustom to different abiotic stresses. For instance, β-amylase1 (BAM1) and α-amylase 3 (AMY3) mediate starch degradation under osmotic stress (Thalmann et al., 2016). The *bam3* mutants efficiently activated starch degradation under osmotic stress conditions (Thalmann et al., 2016). On the other hand, BAM3 and glucan water dikinase (GWD) are effective under cold stress (Thalmann and Santelia, 2017). Moreover, ABA regulates the activity of BAM1 and AMY3 in leaves under osmotic stress via the AREB/ABF-SnRK2 kinase-signaling pathway (Thalmann et al., 2016). Therefore, ABA-dependent transcriptional coordination and differential regulation of starch metabolism is critical for abiotic stress response (optimal energy supply under stress conditions) in cereals (Mukherjee et al., 2015; Thalmann and Santelia, 2017).

Several TFs directly regulate starch biosynthesis (see (Li et al., 2021b; Li et al., 2021c)). Meanwhile, the protein kinase sucrose non-fermenting1 (SNF1)-related kinase 1 (SnRK1) is activated when energy levels decline during stress, reconfiguring starch metabolism and gene expression to favour carbon degradation than build-up, ultimately restoring energy balance and homeostasis (Peixoto and Baena-González, 2022). Therefore, the capacity to efficiently redistribute resources is essential for plants to cope with abiotic stress, hence; targeted manipulations that enhance SnRK1 activity and alter central metabolism may yield improved abiotic stress tolerance in crops (Peixoto and Baena-González, 2022). We opine that using modern tools such as single cell transcriptomics (Fiers et al., 2018; Xia et al., 2022) and machine learning (ML) (Cortés and López-Hernández, 2021; Sidak et al., 2022) to uncover the complex starch biosynthesis regulatory networks, and the less explored enzymes and genes (including TFs) (Cho and Kang, 2020; Huang et al., 2021) will pave way for the identification of novel alleles and targets (core/hub genes and key pathways) for manipulation (eg., via OE of multiple pathway enzymes (Li et al., 2021c) using CRISPR-Cas9 (Gao, 2021; Shelake et al., 2022) to enhance leaf and endosperm starch capacity, optimize energy use efficiency, and improve abiotic stress tolerance in cereals (Cho and Kang, 2020; Huang et al., 2021).

### 4.3 GABA (γ-aminobutyric acid) biosynthesis

GABA is a ubiquitous non-protein amino acid which is conserved across animal, plant and bacteria kingdoms (Shelp et al., 1999; Bouché and Fromm, 2004; Shelp et al., 2012; Bown and Shelp, 2016). Whereas its cellular communication functions are well documented in animals, GABA`s physiological and molecular roles in plants have recently emerged (Fromm, 2020; Hasan et al., 2021; Khan et al., 2021; Li et al., 2021a). GABA is synthesized in the cytosol through the GABA shunt pathway, bypassing two stress inhibited reactions of the mitochondrial-localized tricarboxylic acid (TCA) cycle (Michaeli and Fromm, 2015; Bown and Shelp, 2016; Li et al., 2021a). GABA biosynthesis can also possibly occur via the polyamine degradation and proline synthesis routes (Khan et al., 2021). GABA biosynthesis via the GABA shunt pathway involves the direct and irreversible conversion of glutamate to GABA by glutamate decarboxylase (GAD), followed by the reversible transformation of GABA to succinic semialdehyde (SSA) by GABA transaminase (GABA-T), and the subsequent irreversible oxidization of SSA to succinate by SSA dehydrogenase (SSADH). Then, the oxidized SSA (succinate) is catabolized to γ-hydroxybutyrate (GHB) by succinic semialdehyde reductase (SSR) or glyoxylate reductase (GLYR) (Shelp et al., 1999; Fait et al., 2008; Shelp et al., 2012; Mei et al., 2016; Khan et al., 2021). GABA production in plants is up-regulated by stress, and GABA is fed back into the TCA cycle to maintain cellular energy production (Michaeli and Fromm, 2015; Sita and Kumar, 2020; Xu et al., 2021a). Therefore, GABA shunt components have a vital role of maintaining ion homeostasis and abiotic stress tolerance [reviewed in (Khan et al., 2021)].

Essentially, GABA rapidly accumulates during plant responses to abiotic and pathogenic and insect attacks (Bown and Shelp, 2016), and elevated GABA concentrations invigorate plant stress tolerance by enhancing photosynthesis, osmoregulation and antioxidant enzymes activation (Bown and Shelp, 2016; Priya et al., 2019b; Sita and Kumar, 2020; Hasan et al., 2021). Notably, GABA critically modulates metabolic responses to drought (Hasan et al., 2021; Xu et al., 2021a), heat (Li et al., 2019; Priya et al., 2019b), H+D (Li et al., 2018; Zahra et al., 2021), or combined H+L (light) stresses (Balfagón et al., 2022). For instance, GABA signalling modulates stomatal opening to enhance plant WUE and drought tolerance. In Arabidopsis, guard cell GABA synthesis has been found essential and sufficient to minimize stomatal opening and transpirational water loss, thereby improving WUE and drought tolerance, through negative regulation of the guard cell tonoplast-embedded anion transporter (Xu et al., 2021a; Xu et al., 2021b). Meanwhile, exogenously applied GABA significantly improved heat tolerance in *Agrostis stolonifera*, largely by enhancing osmoprotection, photosynthesis capacity and osmotic regulation (Balfagón et al., 2022). Besides, GABA modulates the expression of genes involved in ROS production, signal transduction and stress-responsive processes (Li et al., 2018; Li et al., 2019; Podlešáková et al., 2019). Further, GABA regulates GABA-gated anion channels via the aluminum-activated malate transporters (ALMTs) (Bown and Shelp, 2016; Kaspal et al., 2021) and may be involved in cross talk with phytohormones to activate conserved pathways under stress conditions (Li et al., 2017; Podlešáková et al., 2019; Xu et al., 2021b). Exploring such potential GABA-phytohormones crosstalk is a promising strategy to deliberately alter the GABA biosynthesis pathway for enhancing abiotic stress tolerance in crops. Further, from the foregoing discussion, we postulate that GABA connects primary metabolism to secondary metabolism and physiological processes essential for fine-tuning abiotic stress responses, and, thus, is a prime target for deliberate manipulation to enhance D+H tolerance in cereals.

Thus, the recently discovered roles of GABA in plant stress tolerance (as an essential metabolite, transport regulator and signal transducer) have spurred intensive investigations on its biosynthesis-involved enzymes and genes (Podlešáková et al., 2019; Kaspal et al., 2021; Khan et al., 2021; Xu et al., 2021b). Among the GABA pathway-related enzymes, GAD is the most extensively characterized in plant species, including Arabidopsis (Turano and Fang, 1998), *Camellia sinensis* (Mei et al., 2016) and maize (Zhuang et al., 2010). Therefore, the other enzymes (GABA-T, SSADH, SSR/GLYR, etc) remain unexplored and potential targets for manipulating the GABA pathway for enhancing abiotic stress tolerance in crops (Podlešáková et al., 2019). Additionally, the discovery of putative GABA binding sites and GABA regulation of anion channels (Žárský, 2015) provides further insights into GABA-mediated stress signalling, facilitates further verification, and opens up possibilities for altering GABA-related genes and enzymes for abiotic stress tolerance improvement in cereals (Fromm, 2020; Kaspal et al., 2021). Essentially, emerging tools such as KIPEs3 that facilitate the automatic annotation and analysis of metabolites biosynthesis pathways with great consistence and quality in diverse plant species (Rempel and Pucker, 2022) could potentially drive the identification of not only core biosynthesis players but also candidate genes for bioengineering abiotic stress tolerance and quality improvements in cereals.

Meanwhile, significant alterations to *miR398s*, *aly-miR159c-3p*, *cca-miR156b*, *ama-miR156*, and other novel *miRNAs* (eg. *novel-24223*, *novel-2964*, etc.) engineered GABA-modulated heat tolerance in bentgrass (Li et al., 2019). Additionally, *miRNA396*, *miRNA398*, *miRNA156*, etc. orchestrated heat tolerance in wheat via TF and stress-responsive genes activation (Sihag et al., 2021; Zahra et al., 2021). Besides, *miRNA159* regulates trans-generational stress memory (Stief et al., 2014). Further, miRNAs-mRNAs collaborate to evoke combinatorial effectors in response to co-occurring abiotic stresses (Zhou et al., 2020). Taken collectively, systematic characterization of drought-responsive and heat-responsive ncRNAs, together with elucidation of their gene expression regulation, will facilitate the identification of key hub ncRNAs that can be harnessed for engineering GABA-mediated stress responses and enhance D/+H tolerance in major crops (Hu et al., 2022).

### 4.4 Phenylpropanoid biosynthesis

**Figure 3:**
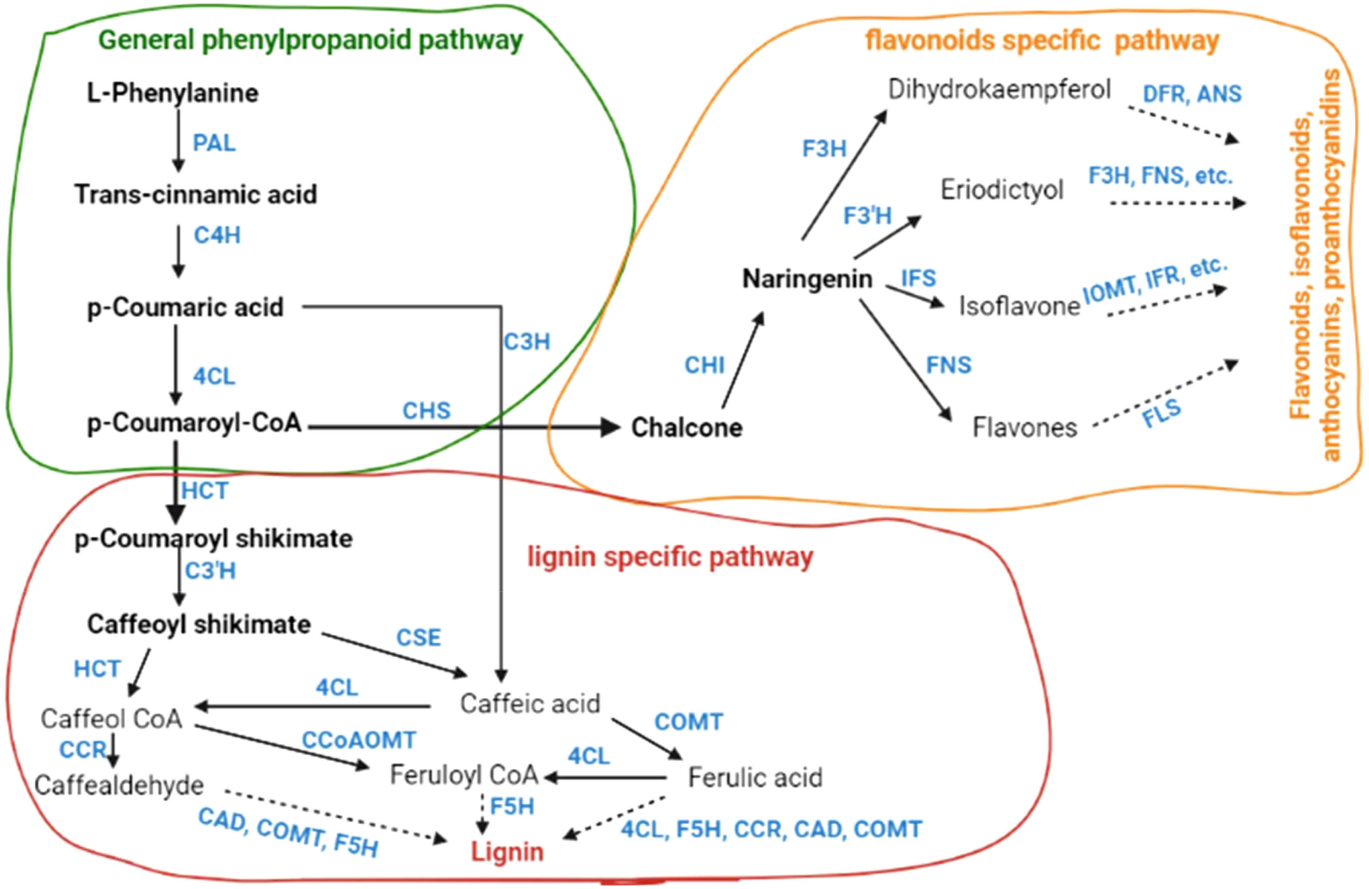
Simplified illustration of the phenylpropanoid biosynthesis pathway and its tributaries. Phenylpropanoid pathway provides precursors to two major downstream routes – lignin and flavonoid biosynthesis pathways, which are the major sources of diverse plant secondary metabolites. Key enzymes are shown in blue and abbreviated as follows: PAL, phenylalanine ammonia lyase; C4H, cinnamate 4-hydroxylase; 4CL, 4-coumaroyl CoA-Ligase; C3H, p-coumaroyl shikimate/quinate 3-hydroxylase; CHS, chalcone synthase; HCT, hydroxycinnamoyl-CoA shikimate/quinate hydroxycinnamoyl transferase; C3′H, p-coumaroyl shikimate 3′ hydroxylase; CSE, caffeoyl shikimate esterase; CCoAOMT, caffeoyl CoA O-methyltransferase; CCR, cinnamoyl CoA reductase; COMT, caffeic acid/5-hydroxyferulic acid O-methyltransferase; F5H, ferulate 5-hydroxylase; CAD, (hydroxy)cinnamyl alcohol dehydrogenase; CHI, chalcone isomerase; F3H, flavanone 3-hydroxylase; FNS, flavone synthase; IFS, isoflavone synthase; FLS, flavonol synthase; IOMT, isoflavone *O*-methyltransferase; IFR, isoflavone reductase; DFR, fihydroflavonol 4-reductase; ANS, anthocyanin synthase; F3′H, flavonoid 3′-hydroxylase. Note: Complete arrows show a one-step enzymatic reaction, whereas dashed arrows denote series of enzymatic steps that have been abstracted for simplicity purposes. Bold text is for emphasis of key stages and/or enzymes. Adopted from (Emiliani et al., 2009; Dong and Lin, 2021; Ferreira and Antunes, 2021).

Anchored by a set of few core intermediates of the shikimate pathway (Herrmann and Weaver, 1999; Fraser and Chapple, 2011), phenylpropanoid biosynthesis pathway generates a variety of specialized metabolites which function in diverse plant growth, development and stress (biotic and abiotic) response processes (Vogt, 2010; Francini et al., 2019; Dong and Lin, 2021). The phenylpropanoid-derived metabolites such as tannins, lignin and suberin provide plant mechanical strength and protection against wounding (Dixon et al., 2002; Cesarino, 2019), heat (Cai et al., 2020; Ren et al., 2021) and drought (Nakabayashi et al., 2014). Several enzyme superfamilies catalyse the pivotal steps of the phenylpropanoid biosynthesis pathway, including ligases, oxygenases, transferases, reductases, oxidoreductases, etc. (Dixon et al., 2002; Vogt, 2010; Fraser and Chapple, 2011); these orchestrate organ-, phenology- and species-specific synthesis of diverse secondary metabolites (Vogt, 2010; Dong and Lin, 2021). Especially, phenylalanine ammonia lyase (PAL), 4-coumaroyl CoA-Ligase (4CL) and cinnamate 4-hydroxylase (C4H) catalyse the mandatory initial three steps of the pathway that provide the basis for all the downstream routes and resultant metabolites (Fraser and Chapple, 2011; Dong and Lin, 2021) (**Figure 3**). PAL modulates the conversion of L-phenylalanine to trans-cinnamic acid by non-oxidative deamination, and directs the subsequent metabolic flux distribution (from the shikimate pathway) to all the downstream branches [for extensive details, see (Kong, 2015)]. The derived metabolites (eg. flavonoids) can then confer stress tolerance, possibly by enhancing antioxidant capacity (Nakabayashi et al., 2014; Nakabayashi and Saito, 2015; Cesarino, 2019; Sharma et al., 2019).

Meanwhile, underpinned by complex gene regulatory networks, the transcriptional regulation of phenylpropanoid biosynthesis exhibits extreme response flexibility to different phenological and stress alterations, which is critical for plant growth and stress adaptation (Francini et al., 2019; Yuan and Grotewold, 2020). Besides, phenylpropanoid biosynthesis is regulated by different signalling pathways and other mechanisms such as post-transcriptional, post-translational, epigenetic and phytohormonal regulations (Dixon and Paiva, 1995; Dong and Lin, 2021). Especially, MYB, WRKYs, NACs and MBW ternary complex TFs regulate the transcription of lignin and flavonoids biosynthesis genes such as *C4H*, *4CL*, *CAD*, *C3H*, *DFR*, *HCT*, *COMT*, etc. in response to abiotic stress (Fraser and Chapple, 2011; Xu et al., 2015; Wessels et al., 2019; Cai et al., 2020; Anwar et al., 2021). For instance, upregulation of *F3H* and *DFR* genes invigorated drought tolerance in Arabidopsis via enhancement of flavonoids (Nakabayashi et al., 2014). Moreover, among the 71 and 11 identified rice heat-responsive DEGs involved in lignin and flavonoids biosynthesis, respectively, most (including *PRX*, *laccase*, *OsPAL*, *Os4CL*, *OsF5H*, *OsF3H*, *OsCHS*, *OsCHI*, etc.) were up-regulated under heat stress, especially in the tolerant genotype SDWG005 (Cai et al., 2020), revealing their crucial role in conferring rice heat tolerance at the meiosis (reproductive) phase. Meanwhile, phenylpropanoid biosynthesis, ROS, and BRs signaling pathways exhibit complex crosstalk in abiotic stress response (Yaqoob et al., 2022).

Here, we underline that leveraging on the advances in comparative- and multi-omics (Scossa et al., 2021; Depuydt et al., 2022; Singh et al., 2022; Zhan et al., 2022), metabolomics (Razzaq et al., 2019; Ma and Qi, 2021; González Guzmán et al., 2022; Hall et al., 2022), single-cell metabolomics (Misra et al., 2014; Fujii et al., 2015; Fiers et al., 2018; Srinivasan and Kannan, 2019; de Souza et al., 2020), computational biology, annotation and analytical (Misra et al., 2014; Ma and Qi, 2021; Perez de Souza et al., 2022; Sidak et al., 2022) approaches that have enabled detection and elaboration of diverse repertoire of trace and specialized metabolites (Hall et al., 2022); we can screen accumulated big data (stored in various databases (Razzaq et al., 2019; Ma and Qi, 2021; González Guzmán et al., 2022)) for key target genes, enzymes and/or metabolites for assembling functional transgenic or synthetic gene regulatory circuits and pathways that can orchestrate enhanced D+H tolerance in cereals (Kong, 2015; Lata and Shivhare, 2021; Zhan et al., 2022). Additionally, PAL, a long-standing target for metabolic engineering of phenylpropanoid biosynthesis (Kong, 2015; Nanda et al., 2017), can be re-engineered using Synbio approaches (García-Granados et al., 2019; Ferreira and Antunes, 2021). For instance, synthetic biosensors (Ferreira and Antunes, 2021), promoters and codon-optimized enzymes (Kong, 2015) can be employed to optimize metabolic fluxes to desired precursor pools or/and inhibit or reduce metabolic fluxes to precursor competitor pathways, thereby enhancing productivity of the target pathway (Kong, 2015; García-Granados et al., 2019). Moreover, synthetic biosensors or regulators can alleviate the challenges of growth inhibition and metabolic burden on the chassis often associated with manipulations to the phenylpropanoid metabolism (Kong, 2015; Muro-Villanueva et al., 2019; Ferreira and Antunes, 2021). Besides, considering the role cell-wall remodelling plays in plant drought and pathogenic resistance (Miedes et al., 2014; Tenhaken, 2014), we can modify root cell-walls (lignification) via phenylpropanoid biosynthesis pathway manipulation to enhance cereal crops tolerance to drought (Yadav et al., 2020; Lata and Shivhare, 2021).

### 4.5 Phytohormone biosynthesis and signalling pathways

**Figure 4:**
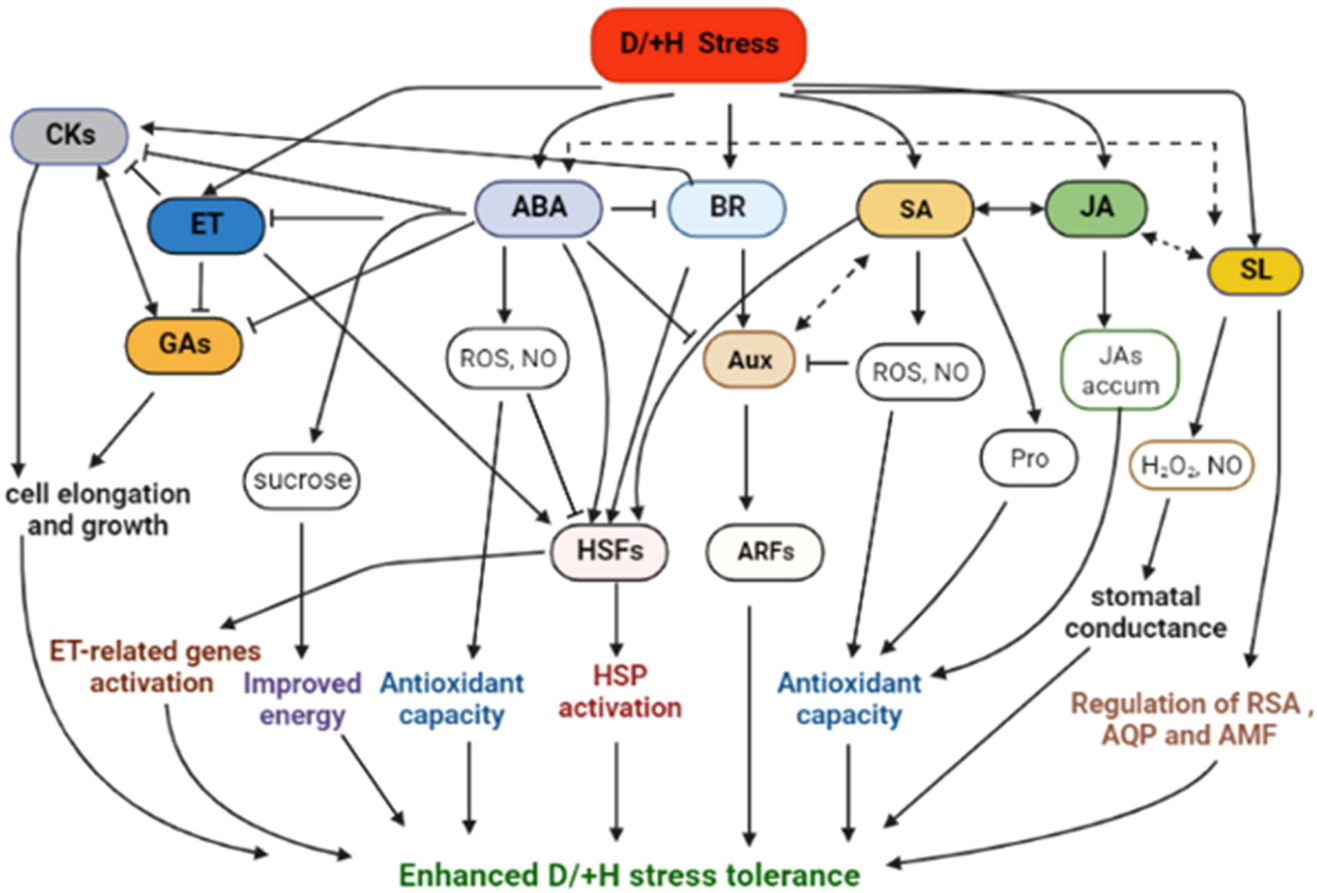
Simplified graphical illustration of the phytohormonal signalling crosstalks underpinning plant drought and/or heat stress (D/+H) stress tolerance. Generally, ABA negatively regulates growth-promoting hormones brassinostroids (BRs), giberrelic acids (GAs), cytokinins (CKs), ethylene (ET) and auxins (Aux). Nonetheless, ABA, BR, ET and salicylic acid (SA) signaling pathways crosstalk to induce D/+H stress tolerance, through sharing similar transcriptional targets (Choudhary and Muthamilarasan, 2022). Meanwhile, SA crosstalk with jasmonic acid (JA) to improve antioxidant capacity, whereas strigolactones (SLs) interact with H_2_O_2,_ nitric oxide (NO) and *SLOW ANION CHANNEL-ASSOCIATED 1 (SLAC1)* to induce stomatal closure and enhance osmotic stress tolerance (Lv et al., 2018). SLs also regulate root system architecture (RSA) adjustment, aquaporins (AQP) activity and AMF processes (Mostofa et al., 2018), which all contribute to enhanced D/+H stress tolerance. Note: For clarity, connections between components have been kept to a minimum; see text for detailed discussion. Solid arrows denote confirmed and positive regulation, whereas blunt ended lines show inhibition. Dashed arrows imply relationships yet to be confirmed. Modified from (Tenorio Berrío et al., 2022; Zenda et al., 2022). Pro, proline; Jas accum, jasmonates accumulation; ROS, reactive oxygen species; HSFs, heat-shock factors; HSPs, ARFs, auxin-responsive factors; heat shock proteins.

Phytohormones such as ABA, BRs, auxins (Aux), ethylene (ET), JA, salicylic acid (SA) and strigolactones (SLs) essentially regulate plant growth and development, as well as environmental stress response (Verma et al., 2016; Mubarik et al., 2021; Choudhary and Muthamilarasan, 2022). Therefore, hormone metabolism and signalling pathways become excellent targets for manipulation to enhance abiotic stress tolerance (Wani et al., 2016; Mellacheruvu et al., 2019; Salvi et al., 2021; Tenorio Berrío et al., 2022). Importantly, phytohormone signal transduction modules crosstalk among themselves (Divi et al., 2010; Verma et al., 2016; Pandey et al., 2017a) and with other stress signalling molecules and modules such as Ca^2+^, ROS, soluble sugars (Rosa et al., 2009; Lv et al., 2018; Jogawat et al., 2021; Salvi et al., 2021; Singh et al., 2022) and MAPK cascades (Li et al., 2020; Salvi et al., 2021), forming a complex network critical for stress response (Jogawat et al., 2021; Choudhary and Muthamilarasan, 2022) (**Figure 4**). Besides, the intricate phytohormone crosstalk essentially modulates transcriptional reprogramming and stress-responsive genes expression (Salvi et al., 2021; Raza et al., 2022b; Zenda et al., 2022).

ABA is the major phytohormone orchestrating drought stress response (Salvi et al., 2021; Choudhary and Muthamilarasan, 2022). ABA signalling pathway regulates DS response via the ABA-SnRK2-PP2Cs-PYLs module (Umezawa et al., 2010; Nakashima and Yamaguchi-Shinozaki, 2013), with SnRK2s phosphorylation of AREB/ABFs being pivotal in ABA-responsive genes expression (Fujita et al., 2013). Plausibly, therefore, ABA biosynthesis and signalling pathways become logical targets for engineering plant drought tolerance (Tenorio Berrío et al., 2022). Overexpression of a *TaABFs*-regulated PYL gene *TaPYL1-1B* improves ABA signalling, photosynthetic capacity and WUE, consequently improving drought tolerance in wheat (Mao et al., 2022). Moreover, modifying the ABA receptors (PYLs) alters ABA signalling, increases WUE, minimises growth arrest whilst enhancing drought tolerance in cereals (Park et al., 2015). Further, the phosphorylation sites of PYL ABA receptors that are the TARGET OF RAPAMYCINE (TOR) kinase targets can be engineered to fine-tune PYLs activity and optimize plant growth and stress response (Wang et al., 2018c). Other targeted modifications to the interplay between core components of the *SnRK2*-*PP2Cs*-*PYLs* module that fine-tune ABA biosynthesis, levels and signalling to optimize the associated ABA-induced growth-productivity trade-offs under stress conditions can be pursued (Bhaskara et al., 2017; Belda-Palazón et al., 2020; Tenorio Berrío et al., 2022).

Meanwhile, WUE can further be enhanced by OE of AQPs, especially the tonoplast- and plasma membrane-intrinsic proteins (Ahmed et al., 2021; Ermakova et al., 2021). For instance, OE of wheat *TaAQP7* enhanced drought tolerance in tobacco (*Nicotiana tabacum*) by improving cellular water retention, ROS homeostasis and antioxidant capacity (Zhou et al., 2012). Interestingly, AQPs are also regulated by various phytohormones including ABA, gibberellins, JA, SA, IAA and CKs (Kapilan et al., 2018; Ahmed et al., 2021). Therefore, on top of modifying ABA biosynthesis, gating of AQPs holds much promise in enhancing D+H tolerance in cereals. Meanwhile, a nuclear-localized *DRIR* (*DROUGHT INDUCED lncRNA*) is significantly induced by ABA treatment, and drought and salt stresses, and positively regulates tolerance to these stresses in Arabidopsis (Qin et al., 2017). The *drir^D^* mutant and DRIR overexpressing transgenic plants exhibited higher sensitivity to ABA, decreased transpirational water loss, and increased tolerance to drought and salinity stresses as compared to Wt plants (Qin et al., 2017). Taken together, modifying ABA biosynthesis, gating of AQPs, and manipulating lncRNAs improves WUE, and hold much promise for enhancing D+H tolerance in cereals.

Besides, TFs such as the NAC, WRKY, DREB2, etc. attune the expression of DS- and HS-responsive genes via the ABA-independent pathway (Lata and Prasad, 2011; Nakashima et al., 2014). For instance, overexpressed *TaWRKY1-2D* confers DS tolerance in transgenic Arabidopsis and wheat, through up-regulated induction of stress-responsive and antioxidant genes such as *AtP5CS1*, *AtRD29A*, *AtCAT1*, *AtPOD1*, *AtSOD (Cu/Zn*), *TaP5CS*, *TaCAT*, *TaPOD*, *TaSOD* (*Fe*), etc. (Yu et al., 2023). Additionally, several components of the ABA-dependent and ABA–independent response pathways crosstalk in stress transcriptional regulation (Nakashima et al., 2014). Other ABA biosynthesis- and catabolism-related enzymes/genes (*NCEDs*, *HvSUS1, HvAGP-L1, HvBAM1, HvBgs, HvABA8’OH-1, HvAO1*, etc.) have been identified (Seiler et al., 2011; Thalmann et al., 2016). We reason that manipulating key components (eg., through OE or repression) or interactions (using specific/conditional promoters) of these intricate signalling networks potentially optimizes ABA biosynthesis-degradation dynamics and enhance D+H tolerance in cereals (Nakashima et al., 2014; Ciura and Kruk, 2018; Tenorio Berrío et al., 2022). For instance, OE of *SNAC* genes (*OsNAC10* and *OsNAC5*) under the control of root-specific (*RCc3*) and constitutive (*GOS2*) promoters enhances drought tolerance via root structural adjustment and improves grain yield in transgenic rice under field conditions (Jeong et al., 2010; Jeong et al., 2013). Deploying diverse drought-responsive tissue- or organ-specific promoters targeting roots or stomata could potentially regulate the expression of pleiotropic genes and help suppress the harmful effects (Nakashima et al., 2014). However, it is worth noting that the resultant effects of those genetic or pathway manipulations will need further verification under field conditions and across spatiotemporal scales (Ciura and Kruk, 2018).

Physiologically, BRs mediate meristematic cell proliferation, cell-wall remodelling and osmolyte accumulation under abiotic stress (Rao and Dixon, 2017; Planas-Riverola et al., 2019; Jogawat et al., 2021; Choudhary and Muthamilarasan, 2022). In fact, BRs and CKs are two key growth promoting hormones that regulate cell division and expansion. However, they are repressed by ABA (Verma et al., 2016). At the transcriptional level, BRs induce HSPs via the BR-dependent TFs such as BRASSINOSTEROID INSENSITIVE 1 (BRI1), BRASSINAZOLE RESISTANT 1 (BRZ1), BRI1-EMS-suppressor 1 (BES1), and phytochrome interacting factors (PIF4, PIF7, etc.). BES1 can also induct the ABA-repressed-PP2Cs facilitated heat shock response pathway [for details, see our recent review (Zenda et al., 2022)]. BRs induce the expression of cyclins (eg. CYCD3;1), cell wall-modifying enzymes and expansins. Additionally, BR signaling activates several TFs such as BIM1, MYB30 and MYBL2 (extensively reviewed in (Planas-Riverola et al., 2019; Tenorio Berrío et al., 2022)). Notably, BR and ABA signaling pathways inhibit each other in abiotic stress responses, converging at the level of brassinosteroid-insensitive 2 (BIN2) and BZR1 (Wang et al., 2018b; Planas-Riverola et al., 2019). On one hand, BIN2 acts a repressor of BR signalling, enhancing ABA-mediated pathway response via phosphorylation of SnRK2, which consequently permit expression of ABA-responsive genes (Cai et al., 2014). Conversely, exogenously applied BR dampens ABA-mediated stimulation of *RESPONSIVE TO DESICCATION 26 (RD26*), a gene encoding a transcriptional activator of stress-inducible gene expression (Chung et al., 2014). This reciprocal antagonism existing between the ABA and BR signalling pathways is critical for plant growth and stress adaptation (Planas-Riverola et al., 2019). Therefore, we can capitalize on this antagonistic relationship to suppress either ABA or BR pathway (depending with the situation) as a strategy to enhance D+H tolerance in cereals. For instance, three BR-signalling-associated TFs (*WRKY46*, *WRKY54* and *WRKY70*) cooperate with BES1 to enhance plant growth, but drought response is dampened through inhibition of drought-inducible gene expression (Chen and Yin, 2017). However, the *wrky46 wrky54 wrky7* tripple mutants exhibit repressed growth and BR levels, but significantly improved drought tolerance (Chen and Yin, 2017). Similarly, OE of the vascular BR receptor *BRL3* enhanced plant drought tolerance (without arresting plant growth) in Arabidopsis through increasing osmoprotectants such as proline, GABA and soluble sugars (Fàbregas et al., 2018). Interestingly, these molecules have already been implicated in D/+H tolerance (Rosa et al., 2009; Pinheiro and Chaves, 2011; El Habti et al., 2020; Xu et al., 2021a; Zahra et al., 2021; Balfagón et al., 2022).

However, any modification to the ABA-BR reciprocal feedback mechanism should take into account the complex crosstalk among ABA, BR and other hormone signalling pathways and molecules. For instance, BRs interact with ET biosynthesis in a way dependent upon BR levels. At low ABA levels, BRs can regulate, via BES1 and BZR1, the suppression of ET biosynthesis, whilst at higher ABA levels, ET biosynthesis is enhanced post-transcriptionally (Jiroutova et al., 2018; Tenorio Berrío et al., 2022). Besides, the observed BR-mediated heat and salinity tolerance in Arabidopsis has pointed to a possible cross-talk of BR with SA, ABA and ET signaling pathways, through sharing similar transcriptional targets (Divi et al., 2010) (**Figure 4**). All these interconnectivities, when not carefully considered in the engineering experiment design, may have negative effects on the actual intended metabolic pathway improvement.

Meanwhile, strigolactones (SLs) together with H_2_O_2_ and nitric oxide (NO) synthesis, and *SLOW ANION CHANNEL-ASSOCIATED 1 (SLAC1)* activation crucially mediate stomatal closure in ABA-independent manner (Lv et al., 2018). Further, JA, SA, ET have been shown to crosstalk with ABA and AQPs (Khan et al., 2013; Waadt, 2020; Ahmed et al., 2021; Choudhary and Muthamilarasan, 2022; Tenorio Berrío et al., 2022). Here, we advance that decoding complex crosstalk among phytohormone signalling pathways and other signalling molecules and modules, as well as hub components amenable to modification without huge growth penalties, hold much promise to engineering abiotic stress tolerance in cereals. For instance, Schulz et al. (Schulz et al., 2021) employed calcium-dependent protein kinases (CPKs)-mediated combinatorial engineering approach to optimize signalling networks (Ca^2+^ and BR) involved in balancing stress tolerance and growth under water deficit conditions. Targeted genetic transformation of a combination of CPK genes (*CPK28* and *CPK29*) into tobacco enhanced plant tolerance to drought and growth under stress conditions (Schulz et al., 2021). Similar results obtained in proof of concept (Arabidopsis) and validation (tobacco) experiments (Schulz et al., 2021) showed that this combinatorial genetic transformation based on synthetic network selection is an innovative and promising approach for engineering complex signalling networks to enhance crop abiotic stress tolerance. Already, deployment of this technique in metabolic pathway engineering in other plant species has been confirmed (Naqvi et al., 2009; Fuentes et al., 2016).

A more recent review highlights how plant hormones such as ABA, JA, SA, BRs, IAA, CKs, etc. interact with neurotransmitters such as melatonin, acetylcholine, etc. to enhance morpho-physiological responses to (and amelioration of) abiotic stress-triggered oxidative stress, by activating the antioxidant system and improving redox homeostasis (Raza et al., 2022b). For instance, melatonin enhances thermotolerance in soybean seedlings through balancing redox homeostasis, orchestrating antioxidant defense, and modulating phytohomones and osmolytes biosynthesis (Imran et al., 2021). Meanwhile, phytohormones can reciprocally modulate epigenetic processes and regulate gene expression via other transcriptional regulatory pathways (Jiang et al., 2023). Therefore, untangling the complex crosstalk existing among phytohormones, stress signalling molecules and osmolytes will crucially assist in pinpointing key hub points or nodes for targeted manipulation for metabolic engineering of D/+H tolerance in crops (Lv et al., 2018; Jogawat, 2019; Jogawat et al., 2021; Saddhe et al., 2021; Sun et al., 2021; Yaqoob et al., 2022). Additionally, a comprehensive understanding of the common features of plant transcriptional- and epigenetic-regulatory pathways and how cross-regulation among phytohormones acts upon gene expression will be key in identifying hub target genes (such as CPKs and MPKs) for engineering D/+H tolerance (Waadt et al., 2022; Jiang et al., 2023; Yin et al., 2023). However, it must be highlighted that engineering phytohormone signalling pathways is challenging and delicate, due to the large genetic redundancy and complexity of the signalling pathways (Braguy and Zurbriggen, 2016). Nonetheless, we envision that the increased and accelerated adoption of advanced Synbio tools such as the genetically encoded phytohormone signaling manipulators (GEPHMans) (Waadt, 2020), synthetic hormone reporters and biosensors (Zhao et al., 2021), genetically encoded aptamers and heterologous systems (Tungsirisurp et al., 2023), coupled with modelling tools, will simplify spatial and temporal monitoring of phytohormones and other analytes such as sugars, helping in the precise identification, analysis and regulation of interconnectivities between multiple pathways (Isoda et al., 2021).

## 5 Perspectives on metabolic engineering targeted approaches associated with D/+H stress tolerance in cereals

Core metabolic pathways related to primary metabolism (viz., carbon metabolism and starch metabolism) and secondary metabolism (viz., phenylpropanoid biosynthesis and GABA biosynthesis), as well as phytohormone biosynthesis and signalling, can be both targeted for manipulation to enhance D/+H stress tolerance as we have shown above. We do appreciate that pathways involved in the biosynthesis of other osmoprotectants such as proline, glycine betine, trehalose, melatonin, and several ROS-scavengers are also critical in abiotic stress tolerance (comprehensively and nicely reviewed in (Khan et al., 2015; Zulfiqar et al., 2019; Raza et al., 2023a)). However, here, they were not extensively covered; hence, we refer readers to those articles. Rather, our current review majored on the five pathways discussed above. Further, we briefly highlight other perspectives pertinent to improving D+H stress tolerance in cereals, as outlined below.

### 5.1 Multiple-component targeting versus single trait targeting

**Figure 5:**
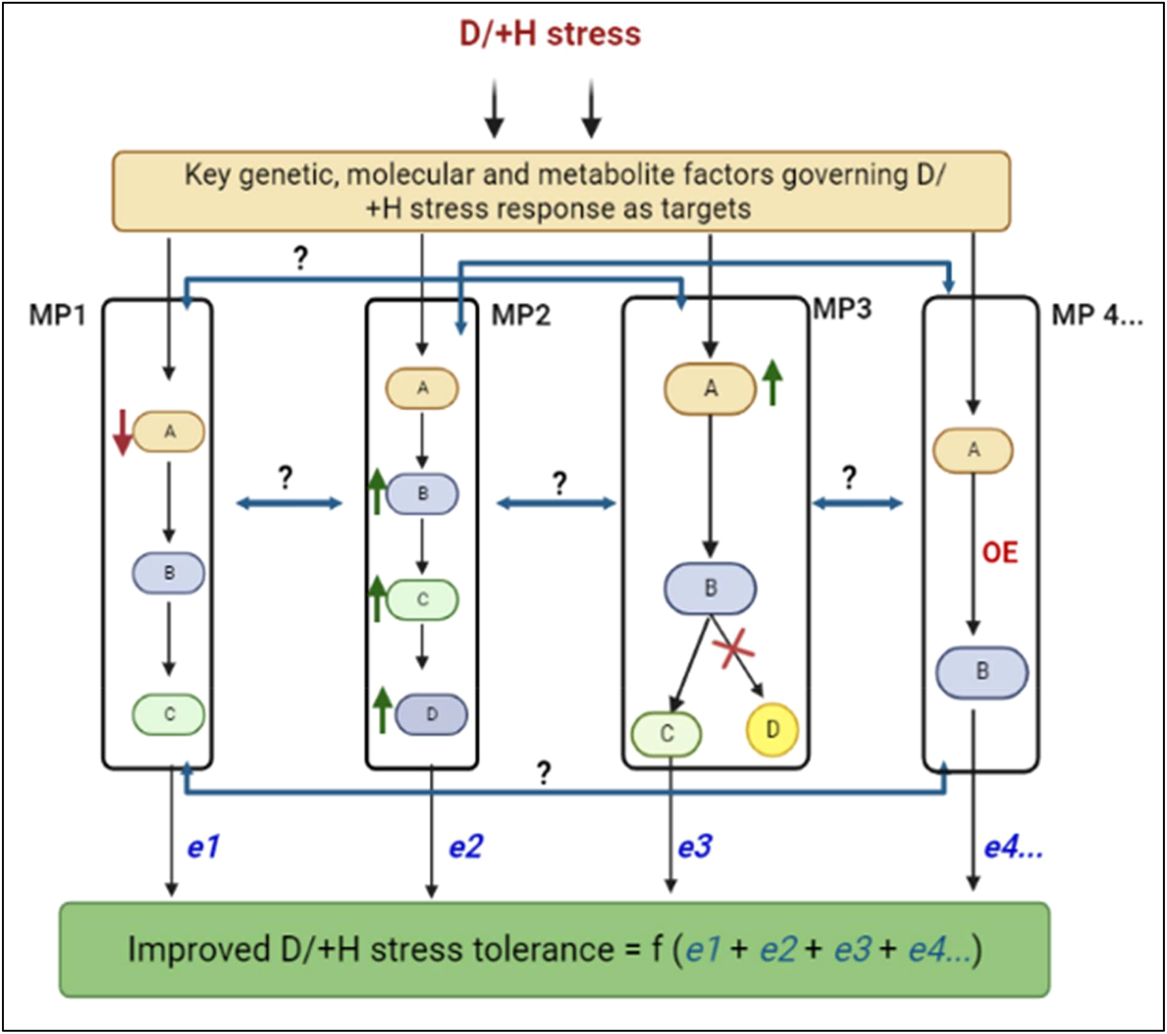
Hypothetical depiction of targeted metabolic pathway manipulation for enhancing drought or/and heat (D/+H) stress tolerance. Description to the diagram: Pods (A, B, C) and (D) denote different genes, numbered sequentially in each pathway for simplicity purposes. Different metabolic pathways are named MP1, MP2, MP3 and MP4. Meanwhile, *e1* to *e4* denote engineered outcome 1 to 4, respectively. Blue connectors with/out questions marks depict crosstalk between pathways, whose effect (synergistic or antagonistic) is either confirmed or unconfirmed. Black pointing arrows between genetic factors depict intermediates (either enzymes, precursors or some metabolites, or series of steps). Green upward pointing arrows show enhancement (overexpression or knock-in) whereas downward pointing red arrows show repression (down-regulation or knock-out). The pathways or arrows or pods are not to scale, but for conceptual depiction only. The depicted concept: Single genetic factor (pod A in MP1) or multiple-gene factors (pods B, C and D in MP2) known to govern D/+H stress response are targeted for modification using modern tools and approaches such as CRIPR-Cas9, synthetic promoters, etc. Genes are either enhanced (OE, knocked-in, etc.), eg., pod A in MP3, or repressed (knocked-down or knocked-out), eg., pod A in MP1. Multiple-genes within the same pathway (eg., B, C and D in MP2) or from different pathways (eg. A in MP3 with any of those in MP2) can be simultaneously expressed. In other scenarios, a competing branch can be blocked to prevent metabolic flux to unwanted or competitor product (branch B-D in MP3), or the intermediate is modified instead of the gene factor (eg. OE of intermediate between A and B in MP4). From these different kinds of manipulations (also explained in **Box 1**), we argue that multiple-gene targeting (either sequentially or simultaneously) from functionally linked pathways harbors great potential to enhance D/+H stress tolerance than single-gene modification (Zhu et al., 2019; Shelake et al., 2022).

We advance that considering the complexity and metabolic pathways or phytohormonal crosstalks related to polygenic traits such as D/+H tolerance, multiple genes/traits or pathways targeting offers more prospects of delivering abiotic stress tolerance versus single-trait modification. This is so because single-trait targeting often leads to unintended consequences on other components or downstream pathways due to feedback regulation (Zhu et al., 2019; Shelake et al., 2022) and stress responses often involve invigoration of overlapping pathways at different levels (Zhang and Sonnewald, 2017). Therefore, simultaneous targeting of multiple genes/components from the same or interlinked pathways potentially eliminates the risk of negative impacts on other system components or whole system functioning and may generate apt responses to D+H stress (Shelake et al., 2022) (**Figure 5**).

### 5.2 Integration: a multi-pronged approach to abiotic stress tolerance improvement

For a multi-pronged cereal crop improvement program for climate resilience and high nutritive value, engineering of key metabolic pathways should be integrated with other plant breeding innovations such as GAB, GS, genomic prediction tools (Crain et al., 2021), ML, AI, multi-omics, speed breeding (Watson et al., 2018), fast forward breeding (FFB) (Varshney et al., 2021a), and smart breeding (Xu et al., 2022). Genomic prediction tools enhances GS, whereas speed breeding and GETs considerably fast-track the development of climate-resilient crops (Tian et al., 2021; Eckardt et al., 2023). FFB integrates advanced genome sequencing, crop phenomics, systems biology, QTL mapping, genomic prediction, ML, AI, and other novel breeding methods to significantly enhance the genetic base of breeding programs and accelerate genetic gains (Varshney et al., 2021a). Meanwhile, smart breeding, driven by Big Data (gathered spatiotemporally over multiple-environmental trial sites), AI, optimized prediction models, and integrated genomic-enviromic prediction (iGEP) (Xu et al., 2022), offers a platform for integration of multi-omics information with ML and AI for targeted designing of breeding-pipelines for crop improvement (Xu et al., 2022). Besides, HT3Ps technologies such as novel sensors and high-resolution imagery can be used to quantify plant performance in specific environments and phenomics data usage optimized for genetic gains (Perez-Sanz et al., 2017; Araus et al., 2018; Crossa et al., 2021; Araus et al., 2022; Hall et al., 2022; Xu et al., 2022). Coupling all these innovations provides the best shot to improving stress resilience in crops (Zenda et al., 2021a; Zenda et al., 2021b; Raza et al., 2023b).

### 5.3 Hitting two birds with one stone: biofortification of stress tolerant cultivars

Based on the successfully deciphered several major QTLs, genomic regions, and genes underlying key nutritive traits in major cereals, including grain zinc (Zn), iron (Fe) and vitamin E contents (Jiang et al., 2017; Zhu et al., 2019; Puranik et al., 2020; Singhal et al., 2021), key metabolic pathways for improving crop grain nutrition quality through biofortification have been identified (Jiang et al., 2017; Zhu et al., 2019; Singh et al., 2021). These pathways include those involved in crop nutrient (especially Fe and Zn) acquisition, uptake and accumulation into grains (Blancquaert et al., 2017; Singh et al., 2021). For instance, enhanced expression of Fe and Zn transporter genes (Blancquaert et al., 2017) or reduced concentrations of anti-nutrient factors (such as phytic acid) (Aluru et al., 2011) enrich Zn and Fe contents [reviewed in (Zhu et al., 2019)]. Simultaneous expression of *FERRITIN* and *nicotianamine synthase* (*NAS*) genes increases both Zn and Fe contents of grains (Wirth et al., 2009; Trijatmiko et al., 2016), whereas concomitant expression of four genes, viz., *FERRITIN*, *NAS*, *carotene desaturase* (*CRTI)* and *phytoene synthase* (*PSY*), yielded a multinutrient-enriched biofortified transgenic rice with greatly enhanced Zn, Fe and β-carotene (caretonoid) contents (Singh et al., 2017). Besides caretonoid, folate, and vitamin E biosynthesis pathways can be targeted for manipulation to enhance grain nutritional contents in cereals such as rice, maize, wheat and pearl millet (Jiang et al., 2017; Kumar et al., 2019; Zhu et al., 2019; Sharma et al., 2021; Singh et al., 2021). For instance, overexpression of *OsMYBR22/OsRVE1* TF significantly and simultaneously enhanced the contents of chloroplast-biosynthesized nutritional and functional metabolites such as carotenoids, chlorophylls, amino acids (lysine and threonine), and amino acid derivatives (eg., GABA) in rice grains; this provides a new strategy for biofortification of rice (Jeong et al., 2022). Essentially, recent advances in metabolomics, plant Synbio and CRISPR-Cas systems have improved our understanding of the biosynthetic pathways, facilitate the reconstruction and regulation of multistep complex metabolic networks, and underpin development of nutrient-dense cereals though biofortification (Zhu et al., 2019). Increased deployment of these strategies in developed elite D/+H tolerant cultivars and other minor cereals across the marginalized drylands, especially the Sub-Saharan African region, will hugely contribute to fighting global malnutrition (Rodríguez et al., 2020).

### 5.4 Challenges and prospects to metabolic engineering for D/+H tolerance improvement

It must be conferred that multiple components or pathway modification for abiotic stress tolerance improvement is not devoid of challenges. The main hurdles evolve around gene and metabolites identification and functional annotation, elucidation of complex crosstalk and redundancy among pathways, metabolomics data analysis, and spatiotemporal analysis of gene expression, among others. Here, we briefly highlight these challenges and the prospects for circumventing them. To start with, D/+H tolerance improvement is on its own complex due to the polygenic nature of these traits, some linkage drag, and the low transformation efficiency and recalcitrance of cereal species (Cortés and López-Hernández, 2021; Silva et al., 2022) (**Box 2**). The identification of precise genes for targeted functions is still a tedious task, especially when dealing with multigenic functions, polygenic traits, or combined stresses (Rivero et al., 2022; Sargent et al., 2022). This is compounded by the lack of information on the functions of several genes and the interactions of different protein family members responding to stress (Shelake et al., 2022), and limited functional annotation capabilities of some bioinformatics tools often used (Zenda et al., 2022). Thus, the key regulators governing D/+H stress tolerance are yet to be conclusively identified. Encouragingly, GWAS (Challa and Neelapu, 2018) and genomic selection (GS) (Meuwissen et al., 2001; Heffner et al., 2009; Xu et al., 2020), coupled with genomic prediction models and phenomics tools (Araus et al., 2018; Crain et al., 2021; Araus et al., 2022; Hall et al., 2022), can help us efficiently identify the major genetic factors underlying D+H stress tolerance. Moreover, third-generation sequencing platforms and their associated long reads (Pucker et al., 2022) now permit high-resolution genome-wide scanning and identification of key genomic regions or haplotypes underlying complex traits such as D/+H tolerance. At the same time, harnessing crop wild relative (CWRs), harbouring untapped diversity for potential climate-responsive traits (Brozynska et al., 2016; Leigh et al., 2022), facilitates incorporation of novel climate-responsive traits/alleles (eg., photosynthetic characteristics, WUE, RSA, floral transition, disease resistance, etc.) into new crop cultivars (Pourkheirandish et al., 2020; Cortés and López-Hernández, 2021). Besides, ML (Sidak et al., 2022) can help us explore the complex regulatory networks and precisely identify core regulators (including novel TFs and protein kinases) mediating abiotic stress responses (Eckardt et al., 2023). Meanwhile, to circumvent the hurdles of low transformation and regeneration efficiencies, as well as genotype dependency of the transformation process in recalcitrant species (including cereals), new methods such as nano particles-based CRISPR-Cas delivery systems, somatic embryogenesis, *de novo* induction of meristem, and transgenic rice endosperms can be used with much better outcome (Demirer et al., 2021; Duan et al., 2021; Chen et al., 2022; Zhu et al., 2022a).

In addition, and more importantly, due to high level complexity, redundancy and crosstalk among several pathways, dissecting the roles of individual elements within those complex networks is more daunting (Braguy and Zurbriggen, 2016; Sargent et al., 2022). Fortunately, Synbio-anchored heterologous orthogonal platforms now help us better understand protein functions and dissect the roles of individual elements/components within complex signalling networks by decreasing the protein environment complexity, minimizing redundancy and limiting interactions with other pathways (Braguy and Zurbriggen, 2016). Moreover, synchronized gene targeting systems (such as TransGene Stacking II; TGSII) premised on site-directed incorporation of transgenes into a genomic position to construct multigene stacks are now feasible (Kumar et al., 2015; Zhu and Liu, 2021). These are mostly mediated by GETs such as CRISPR-Cas 9 (Chen et al., 2019; Zafar et al., 2020; Gao, 2021) and other Synbio tools and approaches (see **Box 2**).

Moreover, due to immense and sheer structural and functional diversity of plant metabolites, identification of key stress-responsive metabolites is yet to be unified (Obata and Fernie, 2012). Particularly, the multi-functionality of secondary metabolites as potent regulators of plant growth, development, stress (biotic and abiotic) defense, and primary metabolites (Erb and Kliebenstein, 2020; Bhambhani et al., 2021) makes it very challenging to precisely disaggregate and analyse the exact metabolites responsible for a particular stress response or phenotype. However, the recent multi-omics-aided discovery of plant metabolic gene clusters has provided new insights into the diversity, evolutionary trajectories, composition, regulation, and function of plant metabolites, which could facilitate efficient pinpointing of desirable metabolites for metabolic engineering of stress tolerance in crops (Weng et al., 2021; Zhan et al., 2022). Meanwhile, mass-spectrometry (MS)-based metabolomics approaches, mostly applied in plant metabolites profiling suffer limitations related to metabolome stochasticity (dynamism), complexity in data analysis (often an opaque of features from both known and unknown metabolites), low sensitivity of analytical instrumentation tools, etc. (Misra et al., 2014; Aretz and Meierhofer, 2016). Besides, cell-localization of metabolites and analysis of spatiotemporal gene expression remain cumbersome (Misra et al., 2014). Consequently, most studies of the interplay between phytohormones and epigenetics, or transcriptional regulation of abiotic stress reported to date have used whole-tissue or single-time-point-collected samples, thereby overlooking spatiotemporal information (Jiang et al., 2023). Here, we advance that the recent advances in MS-based single-cell metabolomics, encompassing microfluidic single-cell cultivation coupled to flow cytometry and advanced MS methods, as well as single-cell analyses, have refined techniques` quantitative abilities, sensitivity, resolution and accuracy at spatial-temporal scales [reviewed in (Jiang et al., 2016; Duncan et al., 2019; Razzaq et al., 2019; Ma and Qi, 2021; Depuydt et al., 2022; González Guzmán et al., 2022; Raza, 2022; Zhou et al., 2022)]. Resultantly, single-cell omics approaches (de Souza et al., 2020; Slavov, 2020; Longo et al., 2021; Mo and Jiao, 2022; Xia et al., 2022), supported by ML and AI (Cortés and López-Hernández, 2021; Sidak et al., 2022), now permit decoding, monitoring and analysis of metabolic fluxes and gene expressions at spatiotemporal scales with greater accuracy (Mo and Jiao, 2022). We anticipate the gradual switch from whole-plant or tissue-level to single-cell level metabolomics, coupled to metabolites databases and other single-omics approaches, to revolutionize the study of metabolic pathways, provide insights into the spatiotemporal interplay among metabolic, epigenetic and transcriptional regulatory networks, and accelerate the development of abiotic stress tolerant crops.

Further, plant growth and development are dynamic processes, and as such, constitutive synthesis of metabolites, by either constitutive overexpression or knock down/out of certain genes or enzymes, may negatively affect (mask or alter) plant cell development and growth (García-Granados et al., 2019; Jiang et al., 2023), On the other hand, metabolic pathway modification may not always translate to predicted or desired outcomes, due to feedback regulation and the complexity of interacting multiple regulators, enzymes and competing pathways (**Figure 5**) (Zhu et al., 2019; da Fonseca-Pereira et al., 2022). Therefore, each targeted modification will require regulators to monitor metabolic flux changes and associated downstream effects. Promisingly, as already highlighted, increased adoption of GEPHMans, synthetic biosensors, and heterologous systems, together with modelling tools, will simplify spatio-temporal monitoring of metabolic flux changes and aid precise identification, analysis and regulation of several crosstalking pathways (Waadt, 2020; Isoda et al., 2021; Zhao et al., 2021; Tungsirisurp et al., 2023). Moreover, these Synbio-based tools have opened up prospects for multiple-traits or pathways modification and creation of novel plant systems custom-designed for specific climate environments (Batista-Silva et al., 2020; Zhu et al., 2020; Shelake et al., 2022; Zhu et al., 2022b). Potentially, this could facilitate translation of most proof of concept discoveries (from lab to field) which have so far remained untested under field conditions, thereby hindering or deferring their incorporation into breeding programs. The limited transferability of metabolic engineering products to ‘outside lab’ environments is largely due to their limited environmental flexibility, and perceived or non-perceived ethical concerns linked to genetically modified organisms (GMOs), which have prompted policy makers to adopt a conservative approach regarding GMO use (Wang and Zhang, 2019; Brooks and Alper, 2021) (**Box 1**). However, an increasing number of countries is reviewing its stance (guidelines and policies) on genome edited products and has shown commitment to international harmonization of policies that promote the future widespread adoption of GMOs (Menz et al., 2020; Liang et al., 2022; Mallapaty, 2022; Sprink et al., 2022; Buchholzer and Frommer, 2023). This, together with trans-boundary multi-disciplinary collaborations among scientists, policy makers, and agricultural extension and communication experts is vital in the promotion and adoption of these new techniques and created stress-resilient and nutrition-enhanced crop cultivars (Emerick and Ronald, 2019; Acevedo et al., 2020).

## 6 Concluding remarks

In view of the current global climate change and the pressing need to sufficiently feed the global human population, alternative strategies for enhancing crop D/+H stress tolerance should be pursued. Designing of those novel strategies relies on first gaining a mechanistic understanding of plant responses to D/+H stress, especially the nature and magnitude of crosstalk among multiple signalling networks. Meanwhile, due to the polygenic and complex nature of D/+H tolerance, and the fast changing climate, single gene targeting approach may not suffice in improving such traits. Conversely, as we opined (abstracted in **Figure 5**) and discussed, metabolic pathways modification holds much promise for effectively improving such complex traits in cereal crops. Strategic targets for manipulation to improve D/+H stress tolerance include carbon, starch, GABA, osmolytes, phenylpropanoid and phytohormonal biosynthesis and signalling pathways as already discussed. Untangling the metabolic circuitry and crosstalk among pathways, and identifying key metabolites and super-coordinated gene expression networks linking primary and secondary metabolism will be critical in future attempts to metabolically engineer D+/H stress tolerance. Additionally, elucidation of the spatiotemporal nature of the stress responsive metabolites and genes is critical. Further, understanding how plant metabolic pathways are regulated facilitates designing of optimized metabolic pathways and precise regulation of metabolic flow for enhanced stress tolerance or nutritional densities. Although metabolic pathway modification is saddled with its own challenges as has been highlighted, the recent advances in molecular biotechnology, single-cell omics, genome editing technologies, computational biology and data analysis approaches, supported by machine learning, offer great opportunities for circumventing these hurdles (Zenda et al., 2023). Especially, we anticipate Synbio-based tools and methodologies such as TGS II and CRISPR-Cas9 to accelerate the development of stress resilient and nutrient-dense cereal crops (as we have proffered in **Box 2**). Besides, single-cell omics approaches, particularly single-cell metabolomics and single-cell transcriptomics, will facilitate for high-resolution single-cell or cell-type-specific identification and quantification of key stress-responsive metabolites and genes, as well as elaboration of spatiotemporal gene expressions, which will aid metabolic engineering for D/+H tolerance in cereals (Depuydt et al., 2022; Hall et al., 2022; Xia et al., 2022; Zhan et al., 2022). Moreover, the gradual shift in policy position on GMOs by an increasing number of countries is a promising move expected to promote the widespread adoption of these Synbio-based methodologies and GMO products, essentially helping in meeting global food security and combating malnutrition.

## Author contributions

SL and ZH conceived the idea. SL, TZ and ZT searched the literature. SL and TZ prepared the original draft manuscript including Figures and Tables. ZH was involved in funding acquisition. All authors contributed to the article and approved the submitted version.
